# Inhibition of c-Rel expression in myeloid and lymphoid cells with distearoyl -phosphatidylserine (DSPS) liposomal nanoparticles encapsulating therapeutic siRNA

**DOI:** 10.1371/journal.pone.0276905

**Published:** 2022-12-15

**Authors:** Christian Bressy, Ali Zemani, Shreya Goyal, Davit Jishkariani, Chin Nien Lee, Youhai H. Chen

**Affiliations:** 1 Department of Pathology and Laboratory Medicine, Perelman School of Medicine, University of Pennsylvania, Philadelphia, Pennsylvania, United States of America; 2 Department of Biological Sciences, University of North Carolina, Charlotte, North Carolina, United States of America; 3 Chemical and Nanoparticle Synthesis Core (CNSC), The University of Pennsylvania, Philadelphia, PA, United States of America; 4 Department of Bioengineering, University of Pennsylvania, Philadelphia, PA, United States of America; 5 Faculty of Pharmaceutical Sciences, CAS Shenzhen Institute of Advanced Technology, Shenzhen, China; Chapman University, UNITED STATES

## Abstract

c-Rel, a member of the nuclear factor kappa B (NF-κB) family, is preferentially expressed by immune cells and is known to regulate inflammation, autoimmune diseases and cancer. However, there is a lack of therapeutic intervention to specifically inhibit c-Rel in immune cells. Recent success with Pfizer and Moderna mRNA lipid-encapsulated vaccines as well as FDA approved medicines based on siRNA prompted us to test a lipid nanoparticle-based strategy to silence c-Rel in immune cells. Specifically, we encapsulated c-Rel-targeting siRNA into distearoyl-phosphatidylserine (DSPS)-containing nanoparticles. DSPS is a saturated phospholipid that serves as the “eat-me” signal for professional phagocytes such as macrophages and neutrophils of the immune system. We demonstrated here that incorporation of DSPS in liposome nanoparticles (LNP) improved their uptake by immune cells. LNP containing high concentrations of DSPS were highly effective to transfect not only macrophages and neutrophils, but also lymphocytes, with limited toxicity to cells. However, LNP containing low concentrations of DSPS were more effective to transfect myeloid cells than lymphoid cells. Importantly, DSPS-LNP loaded with a c-Rel siRNA were highly effective to inhibit c-Rel expression in several professional phagocytes tested, which lasted for several days. Taken together, our results suggest that DSPS-LNP armed with c-Rel siRNA could be exploited to target immune cells to limit the development of inflammatory diseases or cancer caused by c-Rel upregulation. In addition, this newly developed DSPS-LNP system may be further tested to encapsulate and deliver other small molecule drugs to immune cells, especially macrophages, neutrophils, and lymphocytes for the treatment of diseases.

## Introduction

The nuclear factor κB (NF-κB) is a highly conserved eukaryotic transcription factor which has important roles in the regulation of numerous biological processes including inflammation, immune response, developmental processes, cell survival and apoptotic cell death [1–14]. There are five members of NF-κB family identified in mammals: RelA (p65), RelB, NF-κB1 (p105/p50), NF-κB2 (p100/p52) and c-Rel [15]. However, unlike other members of NF-kB family that are ubiquitously expressed, c-Rel is preferentially expressed by immune cells playing roles in inflammation [16–18]. Moreover, c-Rel appears to be frequently amplified in human lymphomas [16,19,20] and is considered as a risk factor for inflammatory auto-immune diseases like psoriasis, encephalomyelitis, rheumatoid arthritis, sclerosis and ulcerative colitis [21–26]. Also, the expression of c-Rel was correlated to the resistance of therapy in these diseases [6,27]. Several studies showed that c-Rel regulates inflammation in immune cells by its ability to induce the expression of cytokines including TNFα, IL-1β, IL-2, IL-6, IL-10, IL-12, IL-15, IL23, IL17A, IFNγ and contributes to create an oncogenic environment by the induction of anti-apoptotic molecules such as Bcl-2, Bcl-XL, Bfl-I and proteins controlling cell survival/cell growth like c-Myc, IRF-4, E2F3a, EP300, GM-CSF, TGFβ, SFN [10,13,28–32]. We recently reported that c-Rel is a key checkpoint for targeted cancer immunotherapy [10]. c-Rel is required for the generation of myeloid-derived suppressor cells (MDSC) by inducing the expression of pro-tumoral genes (such as *Arg1*, *Cebpb*, and *Nos2*) and inactivation of anti-tumoral genes (such as *Il1b*, *Tnfa*, and *Il12p40*). Indeed, c-Rel-deficient mice showed a strong delay in tumor growth. Therefore, c-Rel is a promising therapeutic target for cancer immunotherapy [10].

The conventional approach to reduce inflammation is to use therapeutic molecules such as hydroxyurea, glucocorticoids, azathioprine and methotrexate, However, these systemic treatment strategies are not specific to c-Rel and act on a large variety of pathways in different cells, provoking numerous side effects. More recently, specific c-Rel inhibitors were developed such as IT-603 to block DNA binding of c-Rel protein and its transcriptional activity, or IT-901 to limit the oxidative stress via the inhibition of antioxidant gene such as Heme oxygenase 1 (HMOX1) [33,34]. However, these c-Rel inhibitors have not yet been tested in clinical trials. Another interesting approach is to knockdown c-Rel by RNA interference (siRNA). siRNA uses the capacity of double-stranded RNA molecules, constituted of 20 to 25 base pairs, to enter the cytoplasm of cells and interfere with cellular machinery via the proteins Dicer, Ago and RISC. The gene silencing is induced by siRNA which promote the degradation of the corresponding cellular messenger or the arrest of target protein translation, impairing the expression and the function. Recent studies used a retrovirus or PEG-PLL-PLLeu nanoparticles (Poly (ethylene glycol)-b-poly (L-lysine)-b-poly(L-leucine)) to deliver with success siRNA against c-Rel in lymphocytes B cells [13], macrophages or dendritic cells [22,23,35]. Unfortunately, retroviruses and PEG-PLL-PLLeu nanoparticles are only used in preclinical trials and imposed some drawbacks. For example, retrovirus could be toxic or potentially carcinogenic due to a risk of integration to the host genome. Also, PEG-PLL-PLLeu nanoparticles might have a limited efficacy in humans because they are able to transfect some populations of immune cells like macrophages or dendritic cells but not lymphoid cells [22].

Here we developed a LNP formulation that is able to deliver a siRNA against c-Rel to immune cells with a high efficiency. Liposomes are known for their ability to deliver nucleic acid in cells and induce low cytotoxicity in preclinical and clinical studies. Thus, many liposomes containing siRNA have been developed and used in humans. For example, the Patisiran, a drug based on siRNA/liposome complex and developed by the company Alnylam, was first approved by the FDA in 2018 for the treatment of hereditary transthyretin-mediated amyloidosis [36–39]. The idea was to target with a siRNA a mutated form of the TTR gene, which encodes a dysfunctional transthyretin protein, to prevent formation of the misfolded protein causing diseases in different organs such as heart and brain [36]. A second drug based on siRNA/liposome called Givosiran was developed by Alnylam and approved by FDA in November 2019 to treat acute hepatic porphyria, a genetic disorder provoked by the defect in a liver enzyme, aminolevulinic acid synthase (ALAS1), involved in heme synthesis and accumulation of porphyrin molecules. Givosiran reduced the abundance of the mRNA encoding ALAS1 [40]. In 2020, the FDA approved the third Alnylam’s drug based on siRNA/liposome, called Lumasiran, to treat primary hyperoxaluria type 1 (PH1). This disease is caused by a mutant gene encoding serine-pyruvate aminotransferase, which results in insufficient detoxification of glyoxylate from the liver, resulting in renal and bladder stones and eventually damage to the kidneys. The siRNA/liposome drug reduces the amount of available glyoxylate, a substrate required for the production of oxalate [41]. Inclisiran was the fourth siRNA/liposome drug developed by Alnylam and approved by FDA to be used to reduce low-density lipoprotein cholesterol (LDL-C), the “bad” cholesterol responsible for atherosclerosis [42].

In addition, the efficacy of recent mRNA vaccines, developed by BioNTech/Pfizer and Moderna against Covid-19 and their FDA approval further validated the value of RNA-based drugs. Since transfection of immune cells by nanoparticles (liposome or polymer) is difficult, we chose to incorporate in our LNP a specific lipid, i.e., the distearoyl-phosphatidylserine (DSPS) to engage professional phagocytes like macrophages, neutrophils. Phosphatidylserine (PS) corresponds to a “eat-me-signal” normally present at the surface of apoptotic cells and recognized by specific receptors of phagocytes. The latter cells can recognize apoptotic cells via numerous receptors such as scavenger receptors, PS receptors (Tim proteins, CD300f, Stabilin-2, RAGE, BAI1), integrins (αVβ3, αVβ5), lectins, calreticulin and the complement component 1q (c1q) [43–45]. We found that LNP containing DSPS (DSPS-LNP) increased their uptake in professional phagocytes without provoking cytotoxicity even at high concentrations. Moreover, we demonstrated that the use of a LNP containing 60% of DSPS is sufficient to deliver a fluorescent siRNA with a high efficacy into macrophages and neutrophils. Surprisingly, our DSPS-LNP was also able to transfect lymphocytes, but the delivery of siRNA was less efficient compared to myeloid cells at low siRNA concentration. However, we observed that for a high concentration of siRNA, there was no difference in the siRNA delivery between myeloid and lymphoid cells, indicating that lymphoid cells could also be targeted by our siRNA-based therapy encapsulated by DSPS-LNP. Last but not least, encapsulation of c-Rel siRNA with our DSPS-LNP provoked a strong inhibition of c-Rel expression in professional phagocytes for several days. Taken together, our results suggest that a development of nanoparticle based on DSPS-LNP armed with c-Rel siRNA could improve the targeting of both myeloid and lymphoid immune cells and potentially lead to the elimination of the progression of inflammatory or cancer disease caused by c-Rel.

## Results

### Preparation and characterization of DSPS-LNP to target immune cells

**Fig 1:**
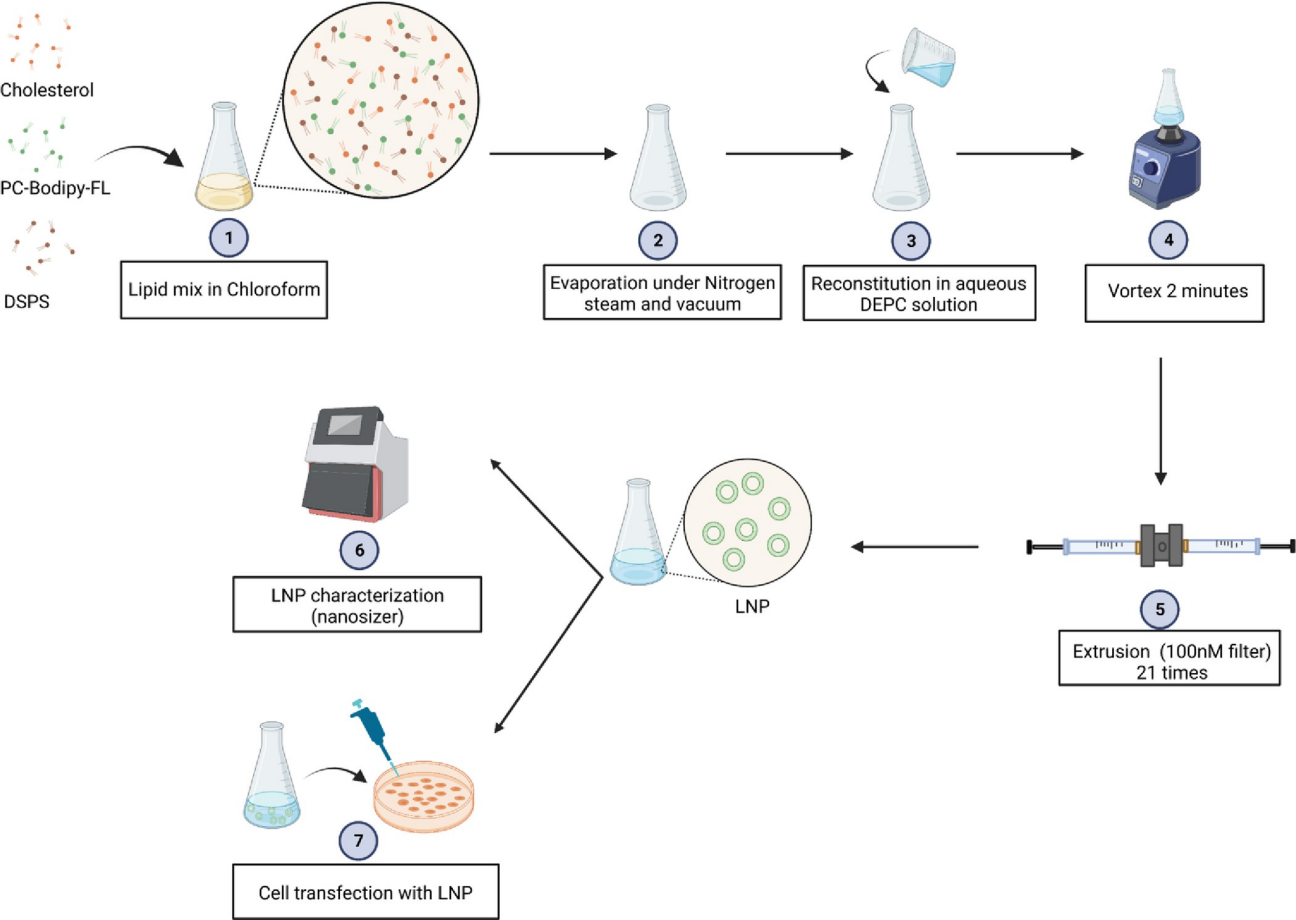
Representation of liposomal nanoparticles (LNP) manufactured. Schematic diagram for the synthesis of DSPS-LNP containing Bodipy-FL with or without cholesterol.

Since our goal was to target immune cells with a high efficiency, we focused on developing LNP with an anionic lipid: the distearoyl-phosphatidylserine (DSPS). Our hypothesis was that DSPS could attract more professional phagocytes such as macrophages and neutrophils, and the negative charge of the LNP would facilitate lymphocyte transfection. To find out the optimal concentration of DSPS to use for immune cells transfection, we constructed five liposomal nanoparticles (LNP) containing different concentrations of DSPS (LNP-S0, LNP-S20, LNP-S45, LNP-S60, LNP-S95 (0 to 95% of DSPS in total lipids)). Besides DSPS, each LNP contained cholesterol (0 to 95% of total lipids) and a constant level of phosphatidylcholine (PC) labeled with a fluorescent probe (Bodipy-FL) (5% of total lipid) to allow LNP tracking by microscopy and flow cytometry **(Table 1** and **Fig 1**). Cholesterol was used not only to adjust the percentage of total lipid to 100% for each LNP but also to allow a better stabilization of LNP as well as facilitation of the lipid nanoparticles fusion with cells.

**Table 1:** Lipid composition of LNP used in this study.

LNP #	% DSPS	% Cholesterol	% PC-Bodipy-FL
1	0	95	5
2	20	75	5
3	47.5	47.5	5
4	60	35	5
5	95	0	5

Composition of five LNP containing different molar ratios of DSPS (1,2-Diacyl-sn-glycero-3-phospho-L-serine), cholesterol and PC-Bodipy-FL (green fluorescent β-BODIPY® FL C12-HPC glycerophosphocholines).

As shown in **S1A–S1C Fig and Table 2**, the constructed LNP showed a size between 102.6 and 124.4 nm, and a polydispersity index (PDI) equal or inferior to 0.1, indicating a homogeneous size for the different LNP made in solution. Moreover, we confirmed with the nanosizer that LNP containing DSPS were negatively charged whereas the LNP without DSPS presented a neutral charge of 0.44 mV.

**Table 2:** Characterization of LNP used in this study.

LNP #	Size average (nm) (±SD)	Poly-dispersity index (PDI) (±SD)	Charge (mV) (±SD)
1	124.4 (±3.95)	0.140 (±0.01)	+0.44 (±0.05)
2	102.6 (±7.66)	0.047 (±0.01)	-5.47 (±0.84)
3	120.8 (±9.11)	0.132 (±0.01)	-16.66 (±2.48)
4	116.4 (±10.05)	0.103 (±0.01)	-28.27 (±4.27)
5	116.0 (±6.18)	0.070 (±0.01)	-48.21 (±5.56)

Each LNP was analyzed by the nanosizer in order to determine the average size in nanometer (nm) as well as the polydispersity index (PDI) and the charge (mV), presented as means ± standard deviations (SD).

### DSPS improves the uptake of LNP in macrophages

**Fig 2:**
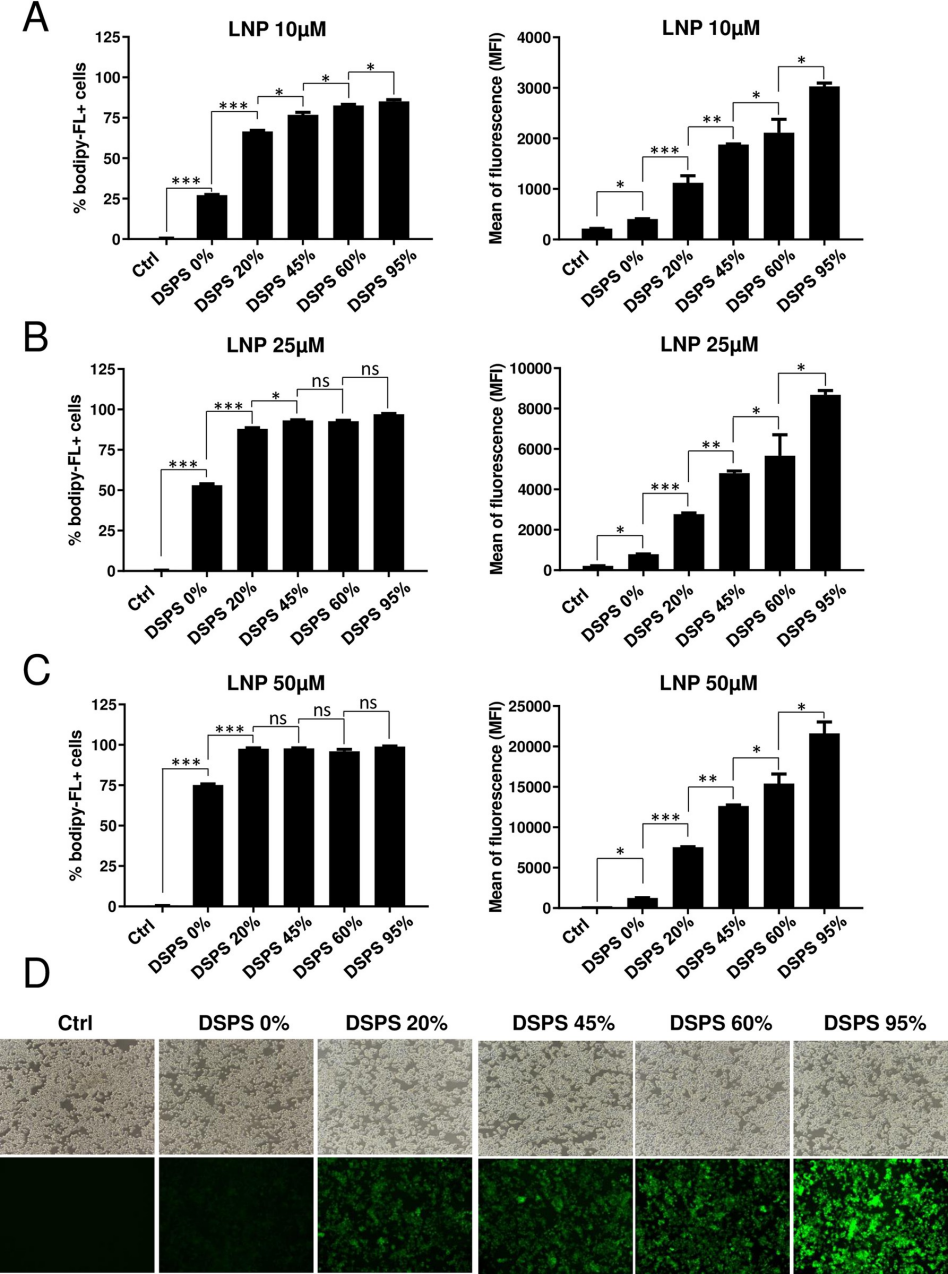
Influence of DSPS on the pickup rate of LNP in macrophage cell line RAW264.7. **(A-C)** RAW264.7 cells (2 × 10^6^) were treated/nontreated with LNP containing 0% to 95% of DSPS at **(A)** 10, **(B)** 25, **(C)** 50 μM, for 24h. The percentage of positive cells (positive for fluorescent probe bodipy-FL) as well as the mean fluorescence intensity were measured by flow cytometry. **(D)** Images of RAW264.7 cells obtained by epifluorescence microscopy at 24h of treatment with LNP at 10μM. The results are representative of two experiments. Data were represented as mean ± standard deviation (SD). Statistical analyses were performed by a One-way ANOVA (with multiple comparisons) complemented by Tukey’s honest significance difference test, and annotated as ns, not significant; *, p < 0.05; **, p < 0.01; ***, p < 0.001.

To investigate whether DSPS affects the pickup levels of LNP by immune cells, we first transfected RAW264.7 murine macrophage cell line with different concentrations of LNP (10μM, 25μM, 50μM) containing various concentrations of DSPS (LNP-S0 to LNP-S95) (**Tables 1 and 3**). After 24 h incubation, the percentage of Bodipy-FL positive cells as well as the mean of fluorescence intensity (MFI) of individual transfection were measured by flow-cytometry (**Fig 2A–2C**). In the absence of DSPS, 10 μM of LNP had the capacity to transfect just ~27% of cells (**Fig 2A left**). In sharp contrast, the percentage of Bodipy-FL positive cells was increased to 66%, 76%, 82% and 85% with LNP containing 20%, 45%, 60% and 95% of DSPS respectively **(Fig 2A left)**. Thus, increasing DSPS concentration in the LNP translated to the higher levels of transfection compared to a non-DSPS LNP and the improvement of the number of transfected cells with DSPS-LNP was statistically significant compared to LNP without DSPS. We also observed DSPS dose-dependent effects in the levels of MFI for 10 μM LNP containing 0%, 20%, 45%, 60% and 95% of DSPS **(Fig 2A right)** and again the results showed statistically significant differences between LNP containing no DSPS and LNP with DSPS. Similar results were obtained with other concentrations of LNP which included 25 μM (**Fig 2B**) and 50 μM (**Fig 2C**). To validate these results, we used epifluorescence microscopy to visualize the transfection efficiency. As shown in **Fig 2D,** RAW264.7 macrophages were transfected with 10 μM of various DSPS-LNP; the intensity of fluorescence Bodipy-FL increased with the raising concentrations of DSPS in the LNP. This indicates that DSPS is able to improve the transfection levels of LNP in RAW264.7 cells. Altogether, our data indicate that DSPS improves the pickup rate of LNP in murine macrophages.

**Table 3:** LNP concentrations.

LNP #	Molar Concentration (μM)	Total Lipid Concentration (μg/ml)
1	0.1 1 10 25 50 200	0.04 0.41 4.12 10.30 20.60 82.40
2	0.1 1 10 25 50 200	0.04 0.48 4.83 12.08 24.17 96.70
3	0.1 1 10 25 50 200	0.05 0.58 5.80 14.50 29.00 116.00
4	0.1 1 10 25 50 200	0.06 0.62 6.25 15.62 31.25 125.00
5	0.1 1 10 25 50 200	0.07 0.75 7.50 18.75 37.50 150.00

Molar concentration (μM) and total lipid concentration (μg/ml) of each LNP used in this study are indicated. Molar concentration indicates the total number of moles of LNP related to the total volume of solution whereas the total lipid concentration indicates the mass of lipids (made by a mixture of different lipid ratio) per volume of solution. For each LNP, an initial stock solution of 200μM was prepared containing 0.2 μmoles of total lipids in 1 ml DEPC-treated RNase-free water. The stock solution was then diluted on cells. Concentrations used in this study are based on molar concentrations.

In the rest of the study, we decided to focus on LNP-S60 (LNP#4), a LNP containing 60% of DSPS and 35% of cholesterol, because we observed that this LNP was able to transfect immune cells such as RAW264.7 cells with the best efficiency at a low concentration (10μM). Although the mean fluorescence of cells transfected with a LNP containing 95% of DSPS was stronger than a LNP containing 60% DSPS in the in vitro studies **(Fig 2A right)**, we decided to choose LNP-S60 that contained cholesterol because it has been reported that the presence of cholesterol in a liposome confers a better stability in vivo notably in the blood of animals after intravenous injection [46]. The long-term objective of our study is to develop a new therapeutical strategy to treat humans. Thus, LNP-S95 which does not contain cholesterol would not be a good choice.

### LNP containing DSPS can reach the cytoplasm of cells without detectable cytotoxicity

**Fig 3:**
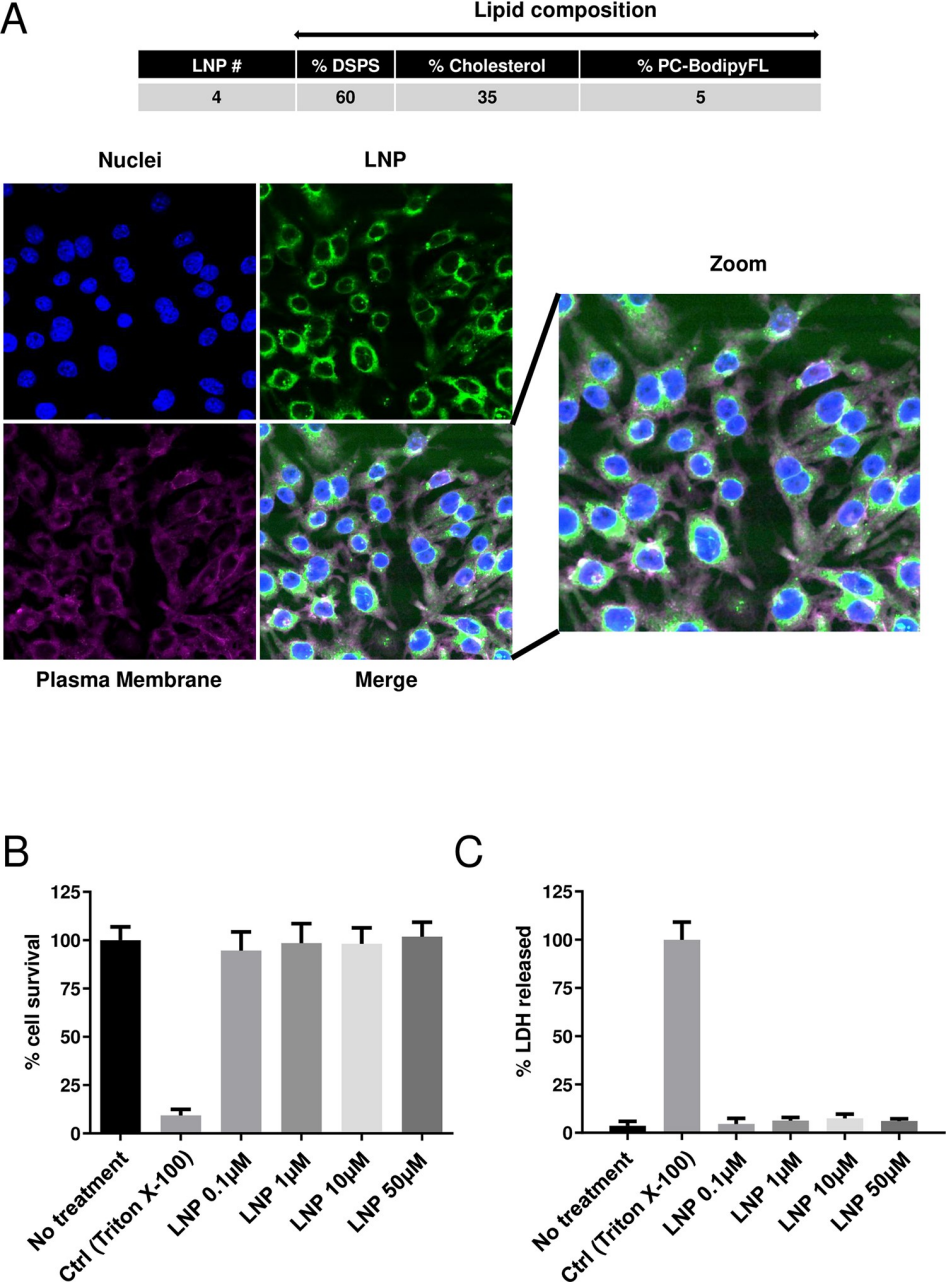
DSPS-LNP can reach the cytoplasm of macrophages without detectable toxicity. **(A)** RAW264.7 cells (2 × 10^6^) were treated for 24 h with LNP-S60 at 50 μM, which contain fluorescent probe Bodipy-FL (green). Cells were then fixed and stained with a fluorescent-labeled WGA coupled with Biotium’s CF®640R (magenta) and DAPI (blue). Cells were imaged by confocal microscopy. **(B-C)** RAW264.7 cells (2 × 10^6^) were treated with different concentrations of LNP-S60 (0 μM, 0.1 μM, 1 μM, 10 μM, 50 μM) or 0.1%Triton X-100 for 72 h, and cell viability was measured by MTT assay **(B)** or LDH assay **(C)**. mean ± SD were expressed relative to none-treated group **(B)** or were expressed relative to the total LDH level obtained from cells treated with 0.1% triton X-100 **(C)**. The results are representative of two experiments.

Next, we investigated the intracellular distribution of LNP-S60 (LNP#4) in RAW264.7 cells after transfection by confocal microscopy. RAW264.7 cells were transfected for 24h with LNP-S60 at 50μM (containing Bodipy-FL (in green)) and then stained with DAPI (in blue) to label nuclei and CF®640R WGA (in magenta) to label plasma membrane. As shown in **Fig 3A**, lipid nanoparticles were not only present at the surface of plasma membrane but also within the cytoplasm. By contrast, we did not find the presence of LNP in the nuclei. These observations indicate that our LNP construct can be used to deliver molecules such as siRNA into the cytoplasm of macrophage cell lines. Moreover, in order to test the toxicity of LNP-S60, we treated RAW264.7 cells with or without different concentrations of LNP (0, 0.1, 1, 10 and 50 μM) for 72 h, and analyzed cell viability by MTT **(Fig 3B)** and LDH assays **(Fig 3C).** As shown in **Fig 3B**, the cell viability was not changed at the concentrations of LNP tested (0.1 to 50 μM). Similarly, insignificant levels of LDH release were detected in the supernatant of cultured cells, indicating that cells remained healthy following our treatment with LNP-S60 (**Fig 3C**). Altogether, we demonstrated that LNP-S60 is safe for cells and can be used for cargo molecule delivery. Finally, we observed no detectable cytotoxicity by MTT (**S2A Fig**) and LDH (**S2B Fig**) assays for other four liposome nanoparticles (LNP) containing different concentrations of DSPS (LNP-S0, LNP-S20, LNP-S45, LNP-S95) confirming that LNP represent an efficient and safe vector for siRNA delivery into cells.

### DSPS-LNP can be used to effectively deliver siRNA to macrophages, neutrophils, lymphocytes and splenocytes

To study whether our LNP-S60 (LNP #4) can be used to deliver siRNA into macrophages and other immune cells, we loaded our Bodipy-FL LNP (LNP-S60) with a siRNA labeled with a red fluorescent probe (cyanine 3) (**S3 Fig**). In a first step, we prepared a stock solution of 200μM of LNP #4 encapsulating 800nM of siRNA and we measured the siRNA encapsulation rate. We got a rate of 89,25% (±3,4) siRNA inside our LNP#4, showing that our LNP was able to entrap a large quantity of siRNA and thus ready to be used for transfection on immune cells.

**Fig 4:**
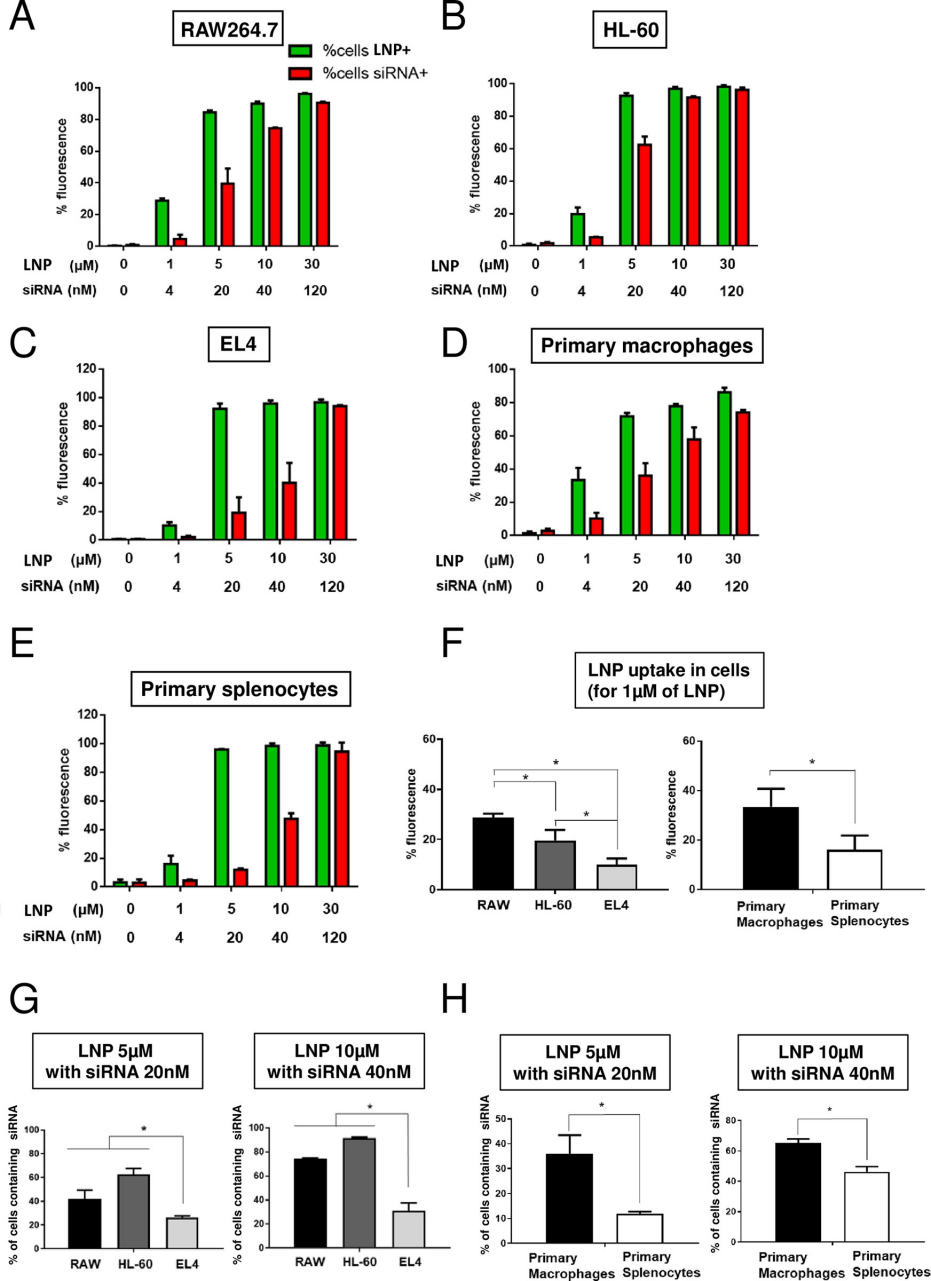
Ability of DSPS-LNP to transfect and deliver fluorescent siRNA into immune cells. 2 × 10^6^ of **(A)** RAW264.7, HL-60 **(B)**, and EL4 cells **(C)**, or primary macrophages **(D)** or splenocytes **(E)**, were seeded in 6-well plates in DMEM containing 10% FBS. On the next day, cells were treated/nontreated with 1 ml LNP-S60 (green fluorescent probe Bodipy-FL) at different concentrations containing fluorescent siRNA (red fluorescent probe cyanine-3) at 37°C in DMEM medium with 10% FBS. The percentage of Bodipy-FL and cyanin-3 positive cells were analyzed by flow cytometry. **(F)** LNP uptake (using 1μM of LNP) was measured in RAW264.7, HL-60, EL4 cells as well as in primary macrophages and splenocytes and compared. Percentage of RAW264.7, HL-60, EL4 cells **(G)** as well as primary macrophages and splenocytes **(H)** containing siRNA was also measured. Results are representative of three independent experiments. mean ± SD were represented. *P <0.05; Two-tailed Mann-Whitney test was used for statistical analysis.

In a next step, different immune cell lines (macrophages (RAW264.7) (**Fig 4A**), neutrophils (HL-60) (**Fig 4B**), lymphocytes (EL4) (**Fig 4C**)) and primary mouse macrophages (**Fig 4D**) and splenocytes (**Fig 4E**), were transfected with LNP-S60-siRNA at several concentrations for 24h. Cells were imaged by epifluorescence microscopy (**S4 Fig**) and the percentage of positive cells (LNP (green) and siRNA (red)) was analyzed by flow cytometry (**Figs 4 and** S5). At the concentration of 1 μM, we observed slightly better transfection rate in RAW264.7 and HL-60 myeloid cell lines compared to EL4 lymphoid cell line (28.8%, 19.6% vs 10.0% respectively) (**Fig 4A–4C and 4F left).** These differences were statistically significant. Nonetheless, for higher concentrations of LNP (between 5 and 30 μM), the percentage of LNP pickup was similar for all three cell types (macrophages, neutrophils, and lymphocytes) and equal or superior to 80% (**Figs 4A–4C, S6A and S6B**). In sharp contrast, whereas the percentage of cells which picked up LNP-S60-siRNA was close between all cell lines, the levels of siRNA delivery showed differences. At all tested concentrations of LNP (1 μM to 10 μM), the percentage of siRNA positive cells was higher in RAW264.7 and HL-60 than for EL4 cells (**Fig 4A–4C and 4G**). Specifically, when we used 5μM of LNP loaded with 20 nM siRNA, the percentage of positive cells was 39% for RAW264.7 and 62% for HL-60 whereas it reached to only 19% in EL4 (**Fig 4G left**). These differences were statistically significant. Likewise, at 10 μM LNP loaded with 40 nM siRNA, the percentage of siRNA positive cells was 74% in RAW264.7 and 91% in HL-60 compared to 40% for EL4 cells (**Fig 4G right**). Here again, these results were statistically significant. However, no difference was observed between the three cell lines, when the highest tested dose of LNP (30 μM) was loaded with 120nM siRNA, showing a transfection efficiency equal or superior to 90% (**S6C Fig**).

In similar experiment, we also transfected primary mouse bone marrow derived macrophages and splenocytes with LNP-S60-siRNA (**Figs 4D, 4E and** S4). The rational to work with splenocytes was that they consist of 80–90% T and B lymphocytes. Interestingly, similar results were observed with these primary cells compared to immune cell lines. As shown in **Fig 4D**, LNP-S60-siRNA were able to transfect, and deliver siRNA into, primary macrophages at the same level as RAW264.7 cells. Splenocytes showed similar levels of transfection efficiency compared to EL4 cells (**Fig 4E**). In addition, we confirmed that our LNP was more effective in transfecting primary macrophages than primary splenocytes with a percentage of uptake reaching 33.4% and 16.05% respectively (**Fig 4F right).** These results were statistically significant. Moreover, as expected, the percentage of siRNA positive cells was improved in primary macrophages (myeloid cells) compared to primary splenocytes (mainly lymphoid cells) (**Fig 4H**). Indeed, we observed that for a LNP armed with a siRNA 20nM, the percentage of cells containing the siRNA was 35.95% for primary macrophages versus 11.95% for primary splenocytes. In the same way, the use of a LNP armed with a siRNA 40nM, the percentage of cells containing the siRNA was 65.12% for primary macrophages versus 46.20% for primary splenocytes. In conclusion, these data indicate that our LNP-S60 was efficient to transfect and deliver siRNA into different immune cells including primary myeloid and lymphoid cell subsets.

### DSPS-LNP loaded with a c-Rel siRNA induced a strong c-Rel gene silencing in professional phagocytes without inducing cell toxicity

**Fig 5:**
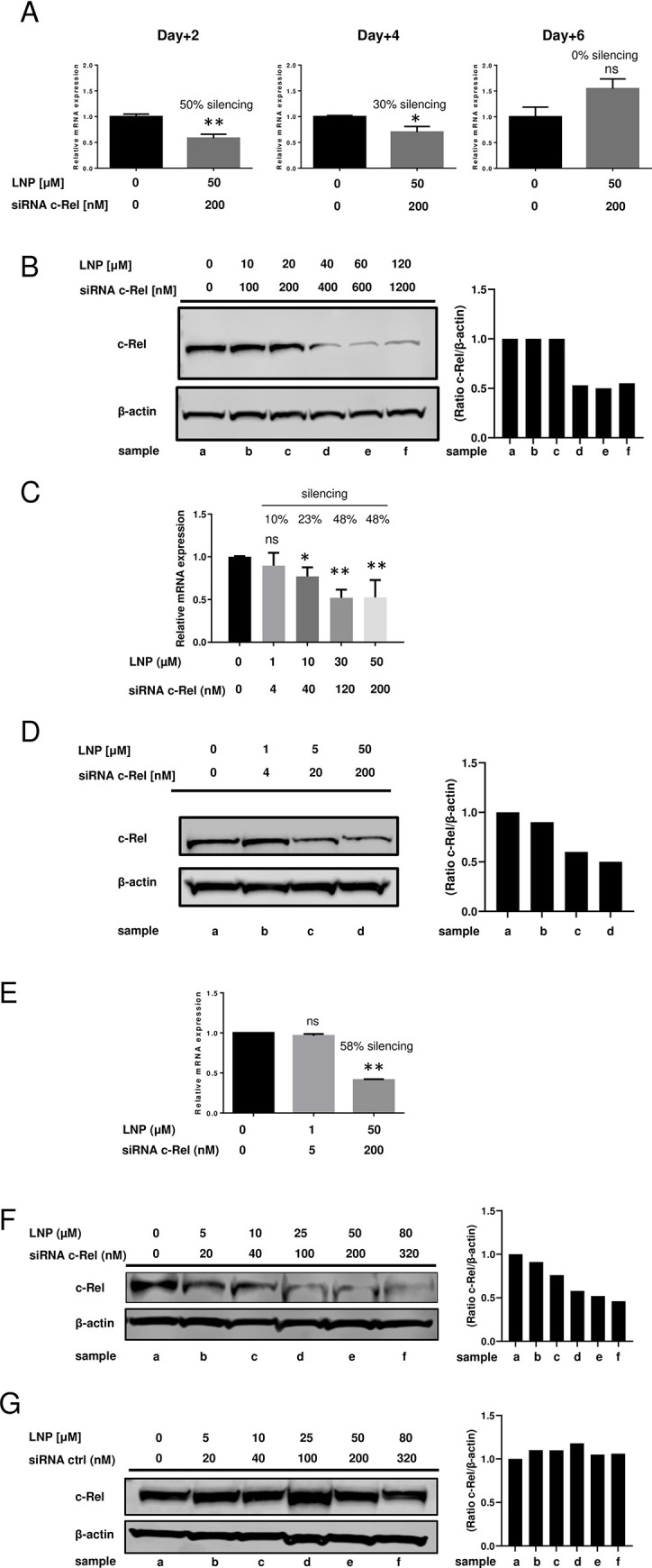
Gene silencing of c-Rel in macrophages with DSPS-LNP encapsulating c-Rel siRNA. **(A)** 2 × 10^6^ RAW264.7 cells were treated/nontreated with LNP-S60 at 50 μM, which contained murine c-Rel siRNA at 200 nM. 2, 4 or 6 days post treatment, the c-Rel messenger RNA expression levels were determined by qRT-PCR. **(B)** The expression of murine c-Rel protein in RAW264.7 cells was measured by western blot 2 days after treatment with LNP (at 0 to 120 μM) encapsulated with murine c-Rel siRNA (0 to 1200 nM). (**C and D**) 2 × 10^6^ HL-60 cells were treated/nontreated with LNP-S60 containing human c-Rel siRNA for 2 days. The human c-Rel messenger RNA **(C)** and human c-Rel protein expression levels **(D)** were determined by qRT-PCR and Western blot, respectively. The level of gene silencing was shown (relative to non-treated group) and results shown are representative of two independent experiments. mean ± SD were represented. *P <0.05; **, P <0.01; ns, nonsignificant. two-tailed unpaired *t* test was used for statistical analysis. 2 × 10^6^ primary macrophages from bone marrow were treated/nontreated with LNP-S60 containing murine c-Rel siRNA **(E and F)** or scramble siRNA **(G),** and the expression of c-Rel mRNA **(E)** as well as the protein **(F and G)** was determined 48 h post transfection. Percentage of gene silencing levels was indicated. Results shown are representative from at least two independent experiments. Mean ± SD were represented. *P <0.05; **, P <0.01; ns, nonsignificant. Two-tailed Mann-Whitney test was used for statistical analysis of the qRT-PCR data. For the Western blot data, a semi quantitative analysis represented by histogram was performed by densitometry. Histogram data was based on the c-Rel/beta actin ratio, indicating the level of c-Rel expression relative to untreated cells.

To silence c-Rel in immune cells, we replaced the fluorescent siRNA within the LNP-S60 with a specific c-Rel siRNA **(Fig 5).** In pilot experiments, we designed a murine c-Rel siRNA sequence (m-c-Rel siRNA) and tested its capacity to provoke the gene silencing in RAW264.7 cells 48 h post siRNA electroporation. We observed a robust inhibition on c-Rel messenger RNA expression by quantitative PCR (**S7A Fig**) using 30 and 300nM c-Rel siRNA (56% and 70% respectively). These results were statistically significant. Next, we tested different concentrations of c-Rel siRNA and evaluated the inhibition level of c-Rel protein in RAW264.7 cells by western blot 48 h post electroporation. We showed that a concentration equal to 250nM of c-Rel siRNA was able to inhibit 60% of c-Rel protein expression (**S7B Fig**). To test the knockdown efficiency of our newly develop LNP-S60. we transfected RAW264.7 cells with or without LNP-S60 at 50 μM containing 200 nM of c-Rel siRNA (LNP-S60-siRNAm-c-Rel) and measured the gene silencing effect by quantitative PCR after 2, 4 and 6-days post-transfection (**Fig 5A**). We observed a 50% suppression on c-Rel messenger RNA following 2 days of initial transfection. Interestingly, the effects of siRNA were also extended until day 4 post treatment with an average silencing of 30%. These results were statistically significant. However, the silencing effect on c-Rel disappeared after 6 days post transfection. In addition, we measured the expression levels of c-Rel protein at 48h post treatment by western blot. We found that expression level of c-Rel protein was not affected at siRNA concentrations less than or equivalent to 200nM. By contrast a concentration of siRNA more than or equivalent to 400nM was effective to suppress the expression of c-Rel protein after 48 h of treatment (**Fig 5B**). Next, we also wanted to know if the gene silencing was attainable in a human HL-60 neutrophil cell line using LNP-S60 containing human c-Rel siRNA (LNP-S60-siRNA hu-c-Rel). We observed a maximal c-Rel gene silencing (48%) with a siRNA concentration ~ 200nM by qPCR (**Fig 5C**) and Western blot (**Fig 5D**). These data are similar to those obtained with RAW264.7 cells. Furthermore, we tested the capability of our LNP-S60-siRNA m-c-Rel to knockdown the c-Rel mRNA and protein expression in primary mouse macrophages. To accomplish this goal, we transfected these cells with 0, 1 and 50 μM of LNP-S60 loaded with c-Rel siRNA at 0, 5 and 200nM respectively and the level of c-Rel mRNA was analyzed by qPCR 48h post transfection (**Fig 5E)**. We found that 1 μM LNP containing 5 nM siRNA was not able to affect the c-Rel messenger level, but a dose of 50 μM LNP loaded with 200 nM siRNA was able to reduce c-Rel expression by 50%. These results were statistically significant. Finally, we checked the inhibition of c-Rel protein by western blot 48h post transfection using different concentrations of LNP containing c-Rel siRNA **(Fig 5F**) or scramble siRNA as control **(Fig 5G**). We observed that LNP at concentrations of 25, 50 and 80 μM armed with c-Rel siRNA at 100, 200 and 320 nM, respectively, were able to significantly inhibit the expression of c-Rel protein in primary macrophages. In contrast, a similar concentration of LNP loaded with scramble siRNA did not show any effect on the level of c-Rel protein expression. Furthermore, we did not observed any cell toxicity measured by both, LDH and MTT assays, even for high concentrations of LNP-S60 (up to 120μM) containing or not c-Rel siRNA (from 0 to 1200nM) on RAW264.7, HL-60, and EL4 cell lines as well as primary cells (splenocytes, macrophages) (data not shown) indicating one more time that our LNP armed with c-Rel siRNA is safe and non-toxic.

Taken together, these results demonstrate that composition of our LNP-S60 containing siRNA against c-Rel is non-toxic and efficient enough to inhibit c-Rel in professional phagocytes and therefore could be further tested in preclinical and clinical studies to counteract the development of inflammation, cancers and other autoimmune diseases that mediated by c-Rel.

## Discussion

Over the past few years, multiple preclinical and clinical studies have shown enhancement of Immune cell function by blocking surface expressed immune checkpoints such as PD1/PDL1/CTLA4; however specific targeting/modulation of intracellular checkpoints at the clinical level has not been achieved so far. Since liposomes has shown a safe means of mRNA delivery (as demonstrated during the COVID-19 pandemic [47–61]), we investigated a LNP formulation that targets one of these intracellular checkpoints (c-Rel) in immune cells.

Nanoparticles have been used in multiple studies to target immune cells and modulate the immune response in inflammation or cancer [62–82]. For example, copolymer of PLGA (poly(lactide-co-glycolicacid)) bearing siRNA against STAT-3 was used to activate dendritic cells and following lymphocyte-T response, avoiding the immunosuppression provoked by the over-expression of STAT-3 [83]. Regarding treatment of inflammatory diseases, some nanoparticles encapsulating siRNA were able to successfully transfect splenocytes and reduce inflammation. For example, liposome CyD1-siRNA/β7 I-tsNPs which was coated with a specific antibody targeting integrin β7 and loaded with siRNA against cyclin D was tested in a model of dextran sulfate sodium (DSS)-induced colitis [84,85]. Other studies have used cationic nanoparticles (PEG-PLL-PLLeu) encapsulating siRNA to inhibit c-Rel expression in arthritis or psoriasis models [22,23,35]. PEG-PLL-PLLeu demonstrated a good silencing effect on c-Rel in macrophages as well as in dendritic cells. However, in these studies, PEG-PLL-PLLeu was not able to target primary lymphocytes. In all listed studies, the main focus was on macrophage and dendritic cells, not other immune cells (neutrophils or lymphocytes) that are also known to control inflammation and chronic immune responses [86,87].

Previous studies on liposome-mediated transfection of lymphocytes using cationic liposomes or polymers have failed [22,74] in preclinical and clinical trials due to the composition of the plasma membrane of lymphocytes which is different from other cells. This has to do with a lack or low levels of negative charged molecules (e.g. heparane sulfate proteoglycans) and overall positive charge in their plasma membrane [74]. This is the main reason that anionic nanoparticle could be more relevant to transfect immune cells in clinical or pre-clinical drug development, especially for the purpose of targeting both myeloid and lymphoid cells.

In this study, we decided to use LNP without polyethylene glycol (PEG), since PEG could reduce the uptake of LNP by neutrophils and other immune cells originated from reticuloendothelial system (RES), such as macrophage [88,89]. Instead, we focused on insertion of a specific lipid, distearoyl-phosphatidylserine (DSPS) into the membrane of the LNP. This lipid is known to be an “eat me” signal present at the surface of apoptotic cells which could attract macrophages. We demonstrated that anionic LNP containing 60% DSPS, i.e., LNP-S60 (along with 35% cholesterol and 5% phosphatidylcholine), were able to effectively transfect several immune cell types including macrophages (RAW264.7 and bone marrow derived macrophage BMDM), neutrophils (HL-60), lymphocytes (EL4) and murine splenocytes (mainly lymphocytes). We found that our DSPS-based LNP were able to transfect, at a similar level, both myeloid and lymphoid cells with a high efficacy. This LNP containing 60% DSPS could interact with the positive charge of the plasma membrane of lymphocytes and therefore, lead to endocytosis of the nanoparticles by the latter cells. In addition, cholesterol (35%) presence in liposome membrane could facilitate the fusion process with the plasma membrane of lymphocytes. Additionally, we showed that replacing PS containing unsaturated 1,2-dioleoyl-sn-glycero-3-phospho-L-serine (DOPS) with DSPS in LNP construct could also influence the uptake level of LNP by immune cells (notably lymphocytes). Importantly, this replacement showed no significant toxicity, even at a high concentration (50 μM). Compared to the PEG-PLL-PLLeu nanoparticle, our LNP construct was able to deliver a fluorescent cyanin-3 siRNA into macrophages (primary macrophages and RAW264.7 cell line) and to neutrophil cell line (HL-60), primary splenocytes and lymphocyte cell line EL4. These results indicate that our liposomal nanoparticles are a good choice for siRNA or potential mRNA delivery into immune cells.

Interestingly, our LNP at moderate concentrations (e.g. 1, 5 and 10 μM) encapsulating siRNA between 4 and 40 nM, showed better cargo delivery in myeloid cells (primary macrophages, RAW264.7, HL-60) than lymphoid cells (EL4) and splenocytes, while the pickup level of LNP was similar between all these cells. One explanation for this observation is that siRNA coupled to the fluorescent probe cyanin-3 could be degraded more quickly in lymphoid cells leading to a decrease of fluorescent signal in these cells. Indeed, these cells are known to be the most difficult for siRNA delivery unlike other immune cell types. One reason for the heightened siRNA degradation in lymphocytes could be related to metabolic differences [74,90]. Importantly, when we used a higher concentration of siRNA (140 nM), we did not observe any differences in siRNA delivery between myeloid and lymphoid cells. Thus, DSPS-based LNP armed with c-Rel siRNA were able to strongly inhibit c-Rel expression in different immune cells at both mRNA and protein levels.

Here, we showed that when we used our LNP with c-Rel siRNA at low concentrations (<100nM), we detected a slight silencing on c-Rel protein in HL-60 cells (see **Fig 5D**) and primary macrophages (see **Fig 5F**). In addition, when we used high siRNA concentrations (>200nM) in our liposome, we improved the gene silencing and observed a dramatic decrease of c-Rel expression in RAW264, HL-60 and primary macrophages. In further experiments, we just used high siRNA concentrations (>200nM) in macrophages to achieve an efficient silencing (≈ 50%). Furthermore, in this study, we tested equivalent conditions between our empty LNP and our LNP containing c-Rel siRNA (for silencing) and we observed no toxicity based on microscopy, trypan blue staining and downstream proliferation assay, even with high concentrations of LNP-S60 (from 10 to 120μM) on RAW264.7, HL-60, and EL4 cell lines as well as primary cells (splenocytes, macrophages) showing one more time that our LNP armed with c-Rel siRNA is safe and non-toxic *in vitro*.

Regarding siRNA concentration, previous studies showed other transfection methods have successfully induced efficient gene silencing in non-immune cells by using much lower siRNA final concentrations (<100nM). As discussed before, most of these transfection reagents are based on cationic lipid-based (e.g. lipofectamine RNAiMax, lipofectamine 2000/3000, interferin, oligofectamine, Fugene), [91–98]. Currently, no studies using lipofectamine or Fugene reagent were reported to transfect efficiently macrophages or lymphocyte to provoke a strong gene silencing. In our study, we also re-tested Lipofectamine and Fugene on immune cells (RAW264.7, HL-60, EL4, primary macrophages) but we were not able to get significant levels of transfection.

Moreover, other studies have tested high siRNA concentrations (200nM, 400nM) to provoke gene silencing in immune cells. For example, Cioca et al [99] used high concentrations of siRNA (400nM) in duplex with Oligofectamine on four different human myeloid leukemia cell lines (HL-60, U937, THP-1, K562). Smith et al [100] used lipid-based reagent DOTAP with 160nM of siRNA on primary human dendritic cells to target TLR7, IRF7 and CXCR4 and Troeger et al used a lipid-siRNA complexes with a HiPerFect transfectant on primary human macrophages with a 200nM siRNA concentration for the gene silencing [101]. However, we also noted in the literature some success of gene silencing obtained with low concentration of siRNA (<100nM) on immune cells *in vitro*: For example, Lipid-Substituted Polyethylenimines were used on THP-1 Acute Myeloid Leukemia cells to silence CXCR4 with 25 to 50 nM of siRNA [102], PEG-PLL-PLLeu micelleplex were used with 100nM siRNA to silence c-Rel in Bone marrow derived dendritic cells (BMDC) and RAW264.7 macrophages to treat Psoriasis [22]. A multifunctional envelope-type nanodevice (MEND) containing YSK12-C4 (YSK12-MEND) were used to transfect efficiently siRNA in human immune cell lines such as Jurkat, THP-1, KG-1, NK92 and provoked gene silencing with low concentrations of siRNA (1 to 30nM) [103]. Finally, a reagent called DharmaFECT3 transfection reagent was also used to transfect and promotes gene silencing in macrophages with low concentrations siRNA (20nM) [104]. However, these reagents cannot be used for human therapy because of their toxicity or cannot be inoculated for practical reasons.

Another efficient method that can transfect immune cells at low siRNA or mRNA concentration is electroporation. Multiple instruments such as Lonza Amaxa or Maxcyte has been developed for these purposes to deliver right amount of electrical pulse to the immune cells. While this method has multiple advantages, however, as electroporation permeabilizes cell membranes, it can compromise cell viability and affect cell function [105–108]. Moreover, electroporation must be conducted *ex vivo* and cannot transfect endogenous lymphocytes, which limits its therapeutic applications [98,109–113].

A major challenge remaining is to show that our LNP armed with a c-Rel siRNA are safe and effective to silence genes in immune cells in vivo, e.g., after an intravenous injection in animals. In addition, the use of thermostable lipid in vivo is extremely difficult, and the process of manufacturing of LNP armed with siRNA is complicated [114]. A strategy developed to overcome this barrier is to use a novel thermostable ionizable lipid-like nanoparticle (iLAND) based on a lead lipid (A1-D1-5) which allows the LNP to be stable at 40°C and to act effectively and safely in hepatocytes for RNAi treatment of hyperlipidemia [114]. However, this novel thermostable ionizable lipid-like nanoparticle (iLAND) based on a lead lipid (A1-D1-5) was not used by the authors to target immune cells such as macrophages, monocytes or lymphocytes where the entrance of nanoparticles is more difficult and the degradation by the endosomes/lysosomes more intense. Another approach to improve the delivery of LNP armed with siRNA in cells and to avoid the degradation by endosome/lysosome system is to use a ionizable lipid called iBL0104 which allows it to escape from endosomes and lysosomes after internalization by the destabilization of their membranes [115]. Again, the toxicity and efficacy studies were done for the liver but not for immune organs or cells [115].

Several strategies have been developed using siRNA-armed nanoparticles to improve cancer immunotherapy [116]. One strategy is to combine siRNA-armed nanoparticles with photodynamic therapy (PDT) which applies photosensitizers (PSs) to create reactive oxygen species (ROS) upon light trigger to destroy cancer cells [117–119]. PDT induces damage in cancer cells causing cell death but also promotes innate immunity, notably by the induction of inflammation, the recruitment of infiltrating leukocytes and the activation of lymphoid cells against tumors. Other studies combined PDT and a nanoparticle armed with PD-L1-targeting siRNA to silence PD-L1 in the microenvironment. The blockage of PDL-1 and PD1 interaction is well known to re-activate the immune system [118,120,121]. A second approach used to improve cancer immunotherapy is to reprogram the immunosuppressive tumor microenvironment by modulating tumor-associated macrophages (TAMs) [116]. For example, nanoparticles armed with siRNA targeting SF1/CSF-1R, which are able to reduce the migration of monocytes to tumors and therefore reduce the number of TAMs, are currently tested in preclinical and clinical studies [116]. All together, these approaches highlighted multiple strategies that cab be taken to improve liposome-based therapy as an efficient non-viral gene transfer vehicle to treat cancer or chronic autoimmune diseases.

In conclusion, our study is the proof of concept that a new formulation of LNP containing 60% of anionic lipids, 35% of cholesterol and 5% of phosphatidylcholine was efficient not only to entrap a siRNA against c-Rel but also efficient to transfect both myeloid and lymphoid immune cells without clear toxicity in these cells. Therefore, DSPS LNP may be used as an efficient non-viral gene transfer vehicle at least in vitro to target multiple genes or signaling pathways in a variety of immune cells while keeping them at high viability rate.

## Materials and methods

### Cell lines and primary cells

Murine macrophage (RAW264.7), murine lymphocyte (EL4) and human neutrophil-like (HL-60) cell lines were obtained from American Type Culture Collection (ATCC) and maintained in DMEM media (10566016, Gibco™, ThermoFisher Scientific) (supplemented with 10% FBS (Life Technologies), 4mM L-glutamine (Cellgro) and 1% antibiotic/antimycotic (Invitrogen, 15240–062).

Primary macrophages were isolated from bone marrow present in femur and tibiae of C57BL/6 mice as previously published. Briefly, primary macrophages were obtained after culture of bone marrow cells during 7 days with DMEM medium (10566016, Gibco™, ThermoFisher Scientific) (supplemented with 10% FBS, 1% antibiotic/antimycotic and 30% L929-cell conditioned medium (LCM). Every 3 days, the old medium was replaced by a fresh medium. Primary macrophages obtained were completely adherent to culture plate and usable on day 8.

Primary splenocytes were also isolated from C57BL/6 mice by straining mashed spleen through a 70-μm cell strainer into ice cold DMEM (Gibco™, ThermoFisher Scientific, 10566016) containing 10% FBS. Cells were spun 1000 g at 4°C for 5 minutes and the supernatant was discarded. The pellet was resuspended with 5 ml ACK Lysing solution (ThermoFisher scientific) for 2 minutes to eliminate red blood cells. Then, 10 ml of DMEM medium supplemented with 10% FBS were added to stop the ACK lysis activity. Cells were then spin down at 1000 g at 4°C for 5 minutes and resuspended in 10% FBS DMEM and used for experiments.

### RNA extraction and qRT-PCR

Total RNA was extracted from cells by trizol-chloroform method. The addition of isopropanol allowed RNA precipitation and RNA pellet was washed with a solution containing 75% ethanol in DEPC-treated water. After centrifugation 7500 g for 5 minutes at 4°C, the RNA pellet was re-suspended in DEPC-treated water and warmed 10 min in a dry bath at 65°C. Final RNA concentration was measured by Nanodrop LITE (ThermoFisher scientific).

1 μg of total RNA were used to converted into cDNA by using the High-Capacity cDNA Reverse Transcription Kit (ThermoFisher scientific, 4368814) according to the manufacturer’s procedure. Specific genes were amplified from cDNA by real time PCR with the Fast SYBR green Master Mix (ThermoFisher scientific, 4385614) using ABI 7300 Real Time PCR system. Relative gene expression levels were calculated as a ratio of the interested gene expression to the housekeeping gene expression (GAPDH or 18S). The following primers were used: murine c-Rel forward (5’-ACCAGAACGCAGACCTTTG-3’) and reverse (5’- TCGCAGTCTTCAATGTCCAG-3’); murine 18S forward (5’-GTAACCCGTTGAACCCCATT-3’) and reverse (5’-CCATCCAATCGGTAGTAGCG-3’); human c-Rel forward (5’ AGAGGGGAATGCGTTTTAGATACA-3’) and reverse (5’-CAGGGAGAAAAACTTGAAAACACA**-**3’); human GAPDH forward (5’- GGTCGTATTGGGCGCCTGGTCACC-3’) and reverse (5’- CACACCCATGACGAACATGGGGGC- 3’).

### Immunoblotting

Whole cell lysate was prepared with RIPA lysis buffer 50 mM HEPES, 0.5M EDTA (pH8.0), 0.5 M EGTA (pH8.0), 1 mM PMSF, 1× complete protease inhibitors mixture (Roche, CO-RO; Merck, 11697498001), and 1× phosphatase inhibitor mixture (Roche, PHOSS-RO; Merck, 4906845001). Protein concentration was determined by BCA assay (ThermoFisher scientific, Pierce™ BCA Protein Assay Kit, 23225). 30 μg of protein sample were electrophoresis in Bolt^TM^ 12% Bis-Tris Plus (Invitrogen) gels and transferred onto a nitrocellulose membrane using electrophoretic transfer system (Invitrogen). The nitrocellulose membrane was blocked in 5% non-fat milk TBS solution at room temperature for minimum 2 hours. Membrane was subsequently probed for either mouse or human c-Rel using 1/1000 dilution of murine monoclonal anti-c-Rel antibody (Santa cruz; G-7; sc-365720) or human polyclonal anti-c-Rel antibody (Cell signaling; #4727) respectively, in 0.1% tween-20 TBS buffer containing 5% non-fat milk at 4°C overnight. The membrane was then washed in TBS-0.1% tween-20 and incubated with HRP-conjugated secondary antibody for 1 hour at room temperature in TBS-0.1% tween-20 with 5% non-fat milk. Finally, Immunoreactivity was visualized using an enhanced chemiluminescence detection kit (ECL, ThermoFisher scientific, Pierce™, 32209). The primary monoclonal antibody anti-β-actin (Sigma, clone AC-15) was used for the detection of mouse and human β-actin. Concerning each Western blot experiment, a semi quantitative analysis using densitometry was performed and results were quantified by Image-J software and expressed as arbitrary units relative to untreated cells.

### Cell viability and death

2 × 10^6^ RAW264.7 cells were plated on 6-well plates for 16–24 hours before treated or un-treated with different concentrations of LNP. After 3 days, cell viability was measured by MTT assay (M5655, Sigma). The results (mean ± SD) were expressed relative to un-treated cell. As a positive control, cells were treated with 0.1%Triton X-100.

To measure the cell death, 2 × 10^6^ RAW264.7 cells were plated on 6-well plates and treated as described above. After 3 days post-treatment by LNP, supernatants were collected, and lactate dehydrogenase (LDH) activity was measured using a Pierce LDH cytotoxicity assay kit (ThermoFisher scientific; 88953). The results were expressed relative to total LDH level obtained from cells treated with identical conditions and permeabilized with 0.1% Triton X-100.

### Bodipy-FL and cyanin-3 positive cell analysis by flow cytometry

2 × 10^6^ RAW264.7, HL-60, EL4 cells, primary macrophages or splenocytes were seeded in 6-well plates in DMEM medium containing 10% FBS. The next day, cells were washed with PBS and treated or untreated with 1 ml LNP (labeled with a green fluorescent probe Bodipy-FL, ThermoFisher scientific, D-3792) at different concentrations containing or not, a fluorescent siRNA (labeled with fluorescent probe cyanine-3, Sigma, SIC003) at 37°C in DMEM medium with 10% FBS. The percentage of positive Bodipy-FL and cyanin-3 cells, and the mean of fluorescence intensity (MFI) were analyzed by flow cytometry on BD LSR Fortessa^TM^ (BD Biosciences) 24 h post-transfection using the fluorescein isothiocyanate (FITC530/30-A) and the cyanine-3 575/26-A) channels.

### Confocal microscopy

RAW264.7 cells (2 × 10^6^) were seeded in Nunc™ Lab-Tek™ II Chamber Slide™ System (ThermoFisher scientific) at 50% confluency and treated with 50 μM LNP (labeled with a green fluorescent probe Bodipy-FL) for 24 h. Cells were washed in PBS and fixed 10 minutes with 3.7% formaldehyde. Next, cells were washed 2 more times with PBS and treated with a plasma membrane stain called Wheat Germ Agglutinin (WGA) labeled with Biotium’s CF®640R dye (ThermoFisher scientific, #29026–1) for 10 minutes at 37°C. After 2 more washing in PBS, cells were stained with DAPI solution for 5 minutes at room temperature. Finally, cells were rinsed by PBS and then dried and imaged with confocal microscope (VT-iSIM) using corresponding filters (excitation/emission) for DAPI (358/461), Bodipy-FL (503/512) and WGA Biotium’s CF®640R (642/662 nm).

### Electroporation

2 × 10^6^ RAW264.7 cells were transfected with different concentrations of murine c-Rel siRNA using a nucleofector II device (Amaxa biosystems) with the Amaxa® Cell Line Nucleofector® Kit V according to the manufacturer’s guidelines. Program Y-001 were used for each electroporation. 48 h after electroporation, total RNA from cells were extracted and the gene silencing of c-Rel was then analyzed by qRT-PCR. 2 μg of pmaxGFP® Vector was used as a control to visualize the quantity of transfected cells (GFP positive) after electroporation with a fluorescent microscope.

### Lipid and siRNA

Cholesterol (C8667) and DSPS (1,2-Diacyl-sn-glycero-3-phospho-L-serine; P6641) were purchased from Sigma Aldrich. Acyl-modified glycerophosphocholines β-BODIPY® FL C12-HPC (2-(4,4-difluoro-5,7-dimethyl-4-bora-3a,4a-diaza-s-indacene-3-dodecanoyl)-1-hexadecanoyl-sn-glycero-3-phosphocholine was purchased from Molecular Probes (D-3792). The nucleotide sequences of c-Rel murine siRNA were 5′-CAACCGGACAUACCCGUCU[dT][dT]-3′(sense) and 5′-AGACGGGUAUGUCCGGUUG[dT][dT]-3′ (antisense). The nucleotide sequences of c-Rel human siRNA were 5′-AAAUGUGAAGGGCGAUCAGCA[dT][dT]-3′(sense) and 5′-UGCUGAUCGCCCUUCACAUUU[dT][dT]-3′ (antisense). A MISSION® siRNA Fluorescent Universal Negative Control #1, Cyanine 3 (SIC003) was also used and purchased from Sigma Aldrich. In addition, scrambled siRNA controls (Sigma Aldrich) are of the same raw composition as the c-Rel siRNA (with same number of bases and same length) but with random orders, so that it does not hybridize with any c-Rel RNA. The nucleotide sequences of scrambled c-Rel murine siRNA were 5′- ACCCCUCUGACAUGGAACC[dT][dT]-3′(sense) and 5′- GGUUCCAUGUCAGAGGGGU[dT][dT]-3′ (antisense).

### Preparation of LNP/siRNA complex

Cholesterol (MP Biomedicals, 0210138225), DSPS (1,2-Diacyl-sn-glycero-3-phospho-L-serine, Sigma, P6641) and glycerophosphocholines (β-BODIPY® FL C12-HPC, ThermoFisher scientific, D-3792) were used at different molar ratio and dissolved in chloroform in a glass tube (total lipid: 0.2 μmoles). Next, the mixture was evaporated under nitrogen gas and dried under a high vacuum for 4 h. The dry lipid layer in the glass tube was hydrated and thoroughly resuspended with 1 ml DEPC-treated RNase-free water with or without siRNA at the desired concentration and then vigorously vortexed for 2 minutes to get a solution of Large Unilamellar Vesicles (LUV) encapsulating siRNA. Afterwards, the solution of LUV/siRNA was sized by extrusion at 21 times through a 100 nm NanoSizer MINI Liposome Extruder (T&T scientific; TT-002-0010) to get the stock and fresh solution of LNP 200μM encapsulating siRNA. LNP and siRNA were incubated for 30 min at room temperature to stabilize the LNP/siRNA complex. The particle size, polydispersity index (PDI) of the LNP and ζ-potential of the LNP were measured by a Zetasizer Nano ZS (Malvern) and analyzed with the Zetasizer software (Malvern). The PDI gives information about the width of the size distribution. A PDI inferior to 0.2 designates a homogenous LNP solution in size. The ζ-potential determined at 37°C gives indications about the charge of the LNP and is expressed in millivolt (the model chosen to calculate the ζ-potential was the Smoluchowski model).

### Measurement of siRNA encapsulation yield in LNP

The efficacy of siRNA entrapment in LNP was determined by a Nanodrop method against a calibration curve. We made empty LNP and LNP containing different concentrations of siRNA. The siRNA loading was firstly calculated by the formulation (Concentration of Total siRNA—Concentration of unentrapped siRNA) and then the yield of encapsulation (%) were calculated according to the formulation: (siRNA loading/ initial siRNA concentration) × 100.

### Statistical analysis

All statistical analyses were performed using GraphPad Prism 7.0a software. We first determined by the Shapiro-Wilk test if our data were normally distributed. For normally distributed data (Figs 2 and S1A), the analysis of variance with an ordinary one-way ANOVA (with multiple comparisons) complemented by Tukey’s honest significance difference test (Tukey’s HSD) was used. Data were represented as mean ± standard deviations (SD). For non-parametric data (Figs 4, 5, **S6** and S7) Mann-Whitney Test (Two-Tailed) was used. Statistically significant differences between groups were annotated as ns, not significant; *, p < 0.05; **, p < 0.01; ***, p < 0.001.

## Supporting information

S1 Fig Determination of size and homogeneity of LNP. Size in nanometer (nm) **(A)** and the distribution of the size by intensity **(B and C)** for each LNP (LNP# 1, LNP# 2, LNP# 3, LNP# 4 and LNP# 5) were measured by a nanosizer with 3 sets of 30 measurements.
(TIF)

S2 Fig Measurement of the toxicity for different LNP manufactured. RAW264.7 cells (2 × 10^6^) were treated with different concentrations of LNP #1, LNP#2, LNP#3, LNP#5 (0 μM, 0.1 μM, 1 μM, 10 μM, 50 μM) or 0.1%Triton X-100 for 72 h, and cell viability was measured by MTT assay **(A)** or LDH assay **(B)**. Mean ± SD were represented relative to none-treated group or were expressed relative to the total LDH level obtained from cells treated with 0.1% triton X-100. The results are representative of two experiments.
(TIF)

S3 Fig Schematic representation of the DSPS-LNP construct, which contained green fluorescent marker (Bodipy-FL) and encapsulated siRNA coupled to red fluorescent probe Cyanine-3. (TIF)

S4 Fig Epifluorescence microscopy image of immune cells after treating with DSPS-LNP encapsulating c-Rel siRNA. 2 × 10^6^ of different immune cell lines **(A)** RAW264.7**, (B)** HL-60**, (C)** EL4**, (D)** primary macrophages and **(E)** primary splenocytes were treated with LNP-S60 (10 μM) containing green fluorescent marker (Bodipy-FL) encapsulated siRNA (40 nM) coupled to red fluorescent probe (Cyanine-3). Cells were imaged 24 h post-treatment.
(TIF)

S5 Fig Uptake measurement of LNP armed with siRNA in different cells by flow cytometry. 2 × 10^6^ of RAW264.7 **(A)**, HL-60 **(B)** and EL4 cells **(C)**, or primary macrophages **(D)** or splenocytes **(E)**, were seeded in 6-well plates in DMEM containing 10% FBS. On the next day, cells were treated/nontreated with 1 ml LNP-S60 (green fluorescent probe Bodipy-FL) at different concentrations containing fluorescent siRNA (red fluorescent probe cyanine-3) at 37°C in DMEM medium with 10% FBS. The cell population was firstly gated (P2 gate) based on Sideward and Forward scattering (SSC-A and FSC-A) and then the percentage of Bodipy-FL and cyanine-3 positive cells were measured in P3 and P4 gates, respectively. In addition, the mean fluorescence intensity (MFI) was also analyzed. Results are representative of three independent experiments.
(TIF)

S6 Fig LNP-S60 uptake and siRNA delivery at different concentrations in RAW264.7, HL-60, and EL4 cells. Different immune cell lines for macrophages (RAW264.7), neutrophils (HL-60), and lymphocytes (EL4) were transfected with LNP-S60 (green fluorescent probe Bodipy-FL) at a LNP concentration of either 5μM (**A**) or 30μM (**B**) for 24h. Then, the percentage of LNP-S60 positive cells was measured by flow cytometry. In addition, the percentage of siRNA positive cells after 24h of transfection with a LNP-S60 (30μM) armed with a siRNA (red fluorescent probe cyanine-3, 120nM) was also analyzed by flow cytometry (**C**). Results were represented by histograms. Results are representative of three independent experiments. mean ± SD were represented. ns, not significant; Two-tailed Mann-Whitney test was used for statistical analysis.
(TIF)

S7 Fig Gene silencing of c-Rel after siRNA electroporation in macrophages. 2 × 10^6^ RAW264.7 cells were electroporated or not with different concentrations of murine c-Rel siRNA for 2 days. Changes in the levels of c-Rel mRNA **(A)** and protein **(B)** were determined. The levels of gene silencing were indicated in red, and results shown are representative of two independent experiments. Mean ± SD were represented. **, P <0.01; ns, nonsignificant. two-tailed unpaired t test was used for statistical analysis. The numbers between the blots (in blue) indicate the level of c-Rel expression relative to untreated cells.
(TIF)

S1 Raw images (PDF)
