# Supplementary figures and images for: Inhibition of c-Rel expression in myeloid and lymphoid cells with distearoyl -phosphatidylserine (DSPS) liposomal nanoparticles encapsulating therapeutic siRNA
Source: PLoS One. 2022 Dec 15;17(12):e0276905. doi: 10.1371/journal.pone.0276905 (PMC9754606; doi:10.1371/journal.pone.0276905)

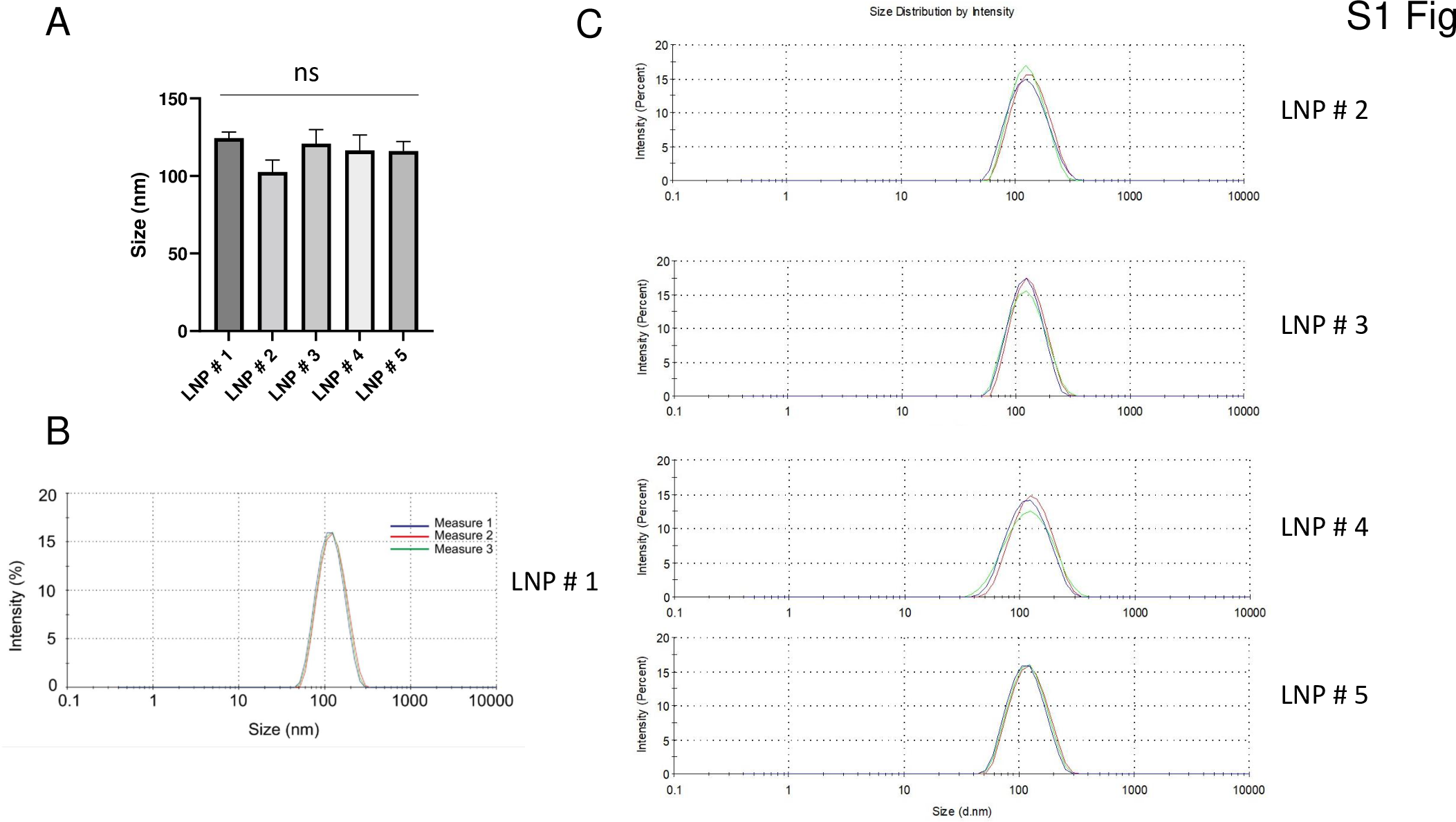

Supplement: S1 Fig — Size in nanometer (nm) (A) and the distribution of the size by intensity (B and C) for each LNP (LNP# 1, LNP# 2, LNP# 3, LNP# 4 and LNP# 5) were measured by a nanosizer with 3 sets of 30 measurements. (TIF) [file pone.0276905.s001.tif]

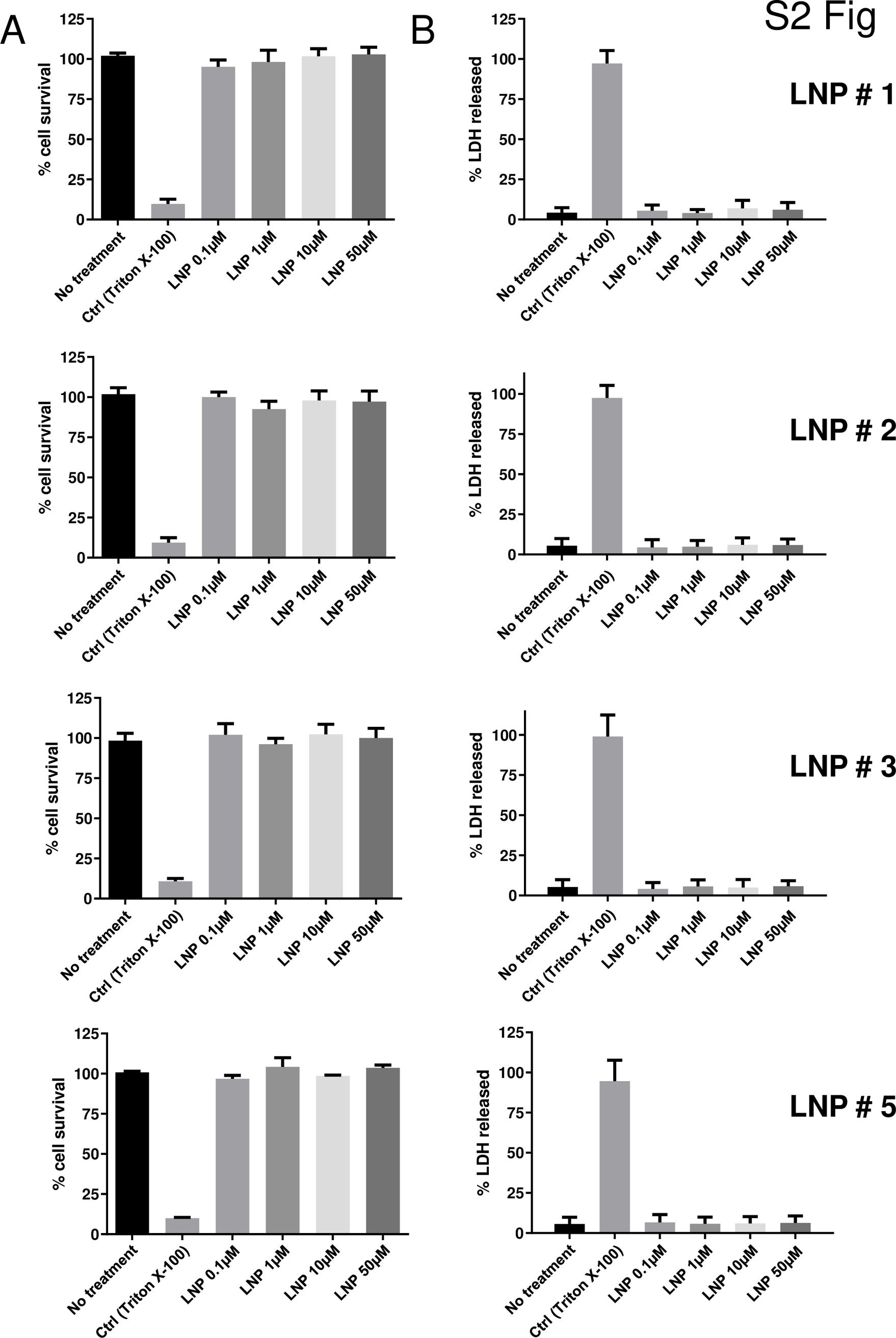

Supplement: S2 Fig — RAW264.7 cells (2 × 10^6^) were treated with different concentrations of LNP #1, LNP#2, LNP#3, LNP#5 (0 μM, 0.1 μM, 1 μM, 10 μM, 50 μM) or 0.1%Triton X-100 for 72 h, and cell viability was measured by MTT assay (A) or LDH assay (B). Mean ± SD were represented relative to none-treated group or were expressed relative to the total LDH level obtained from cells treated with 0.1% triton X-100. The results are representative of two experiments. (TIF) [file pone.0276905.s002.tif]

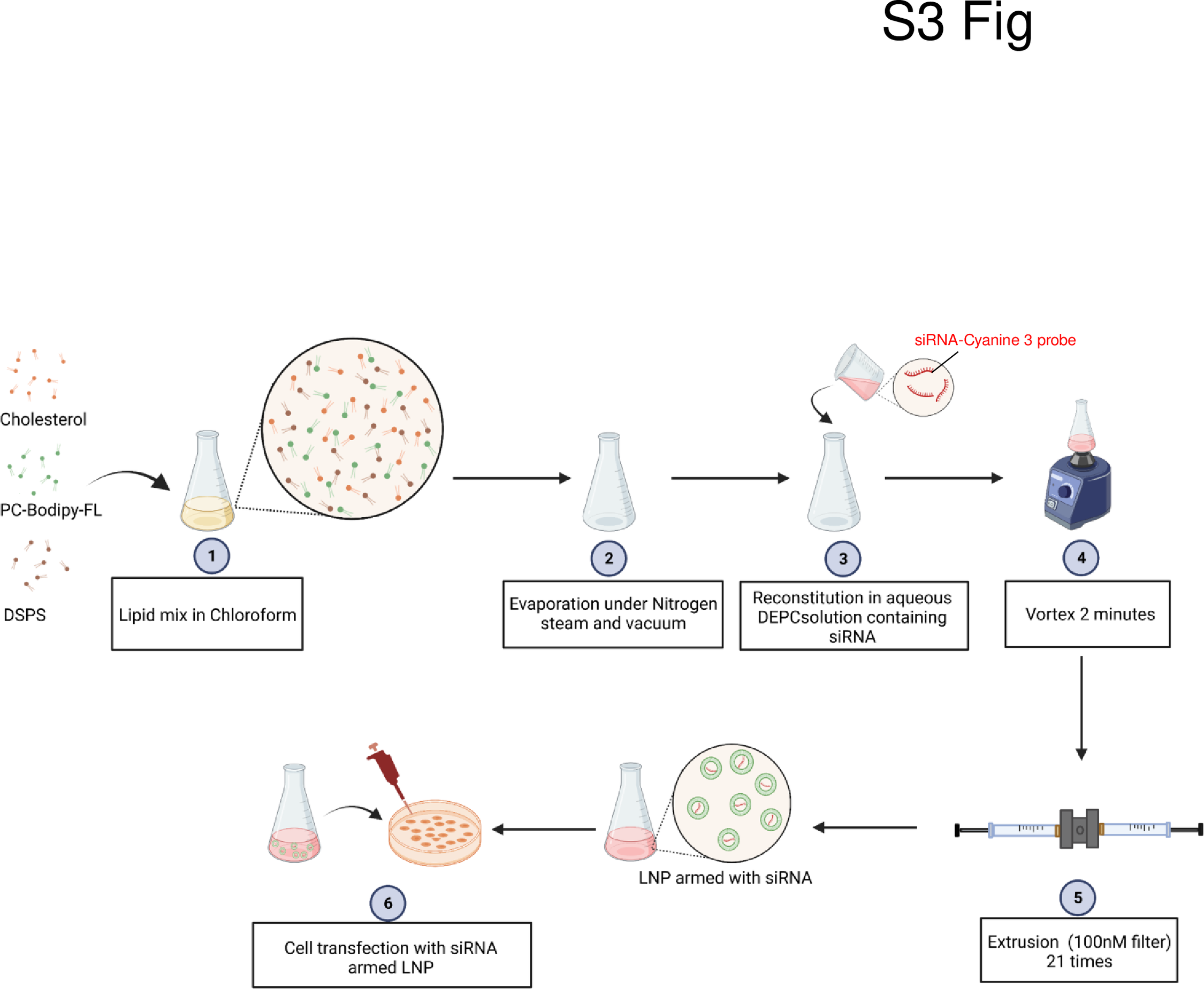

Supplement: S3 Fig — (TIF) [file pone.0276905.s003.tif]

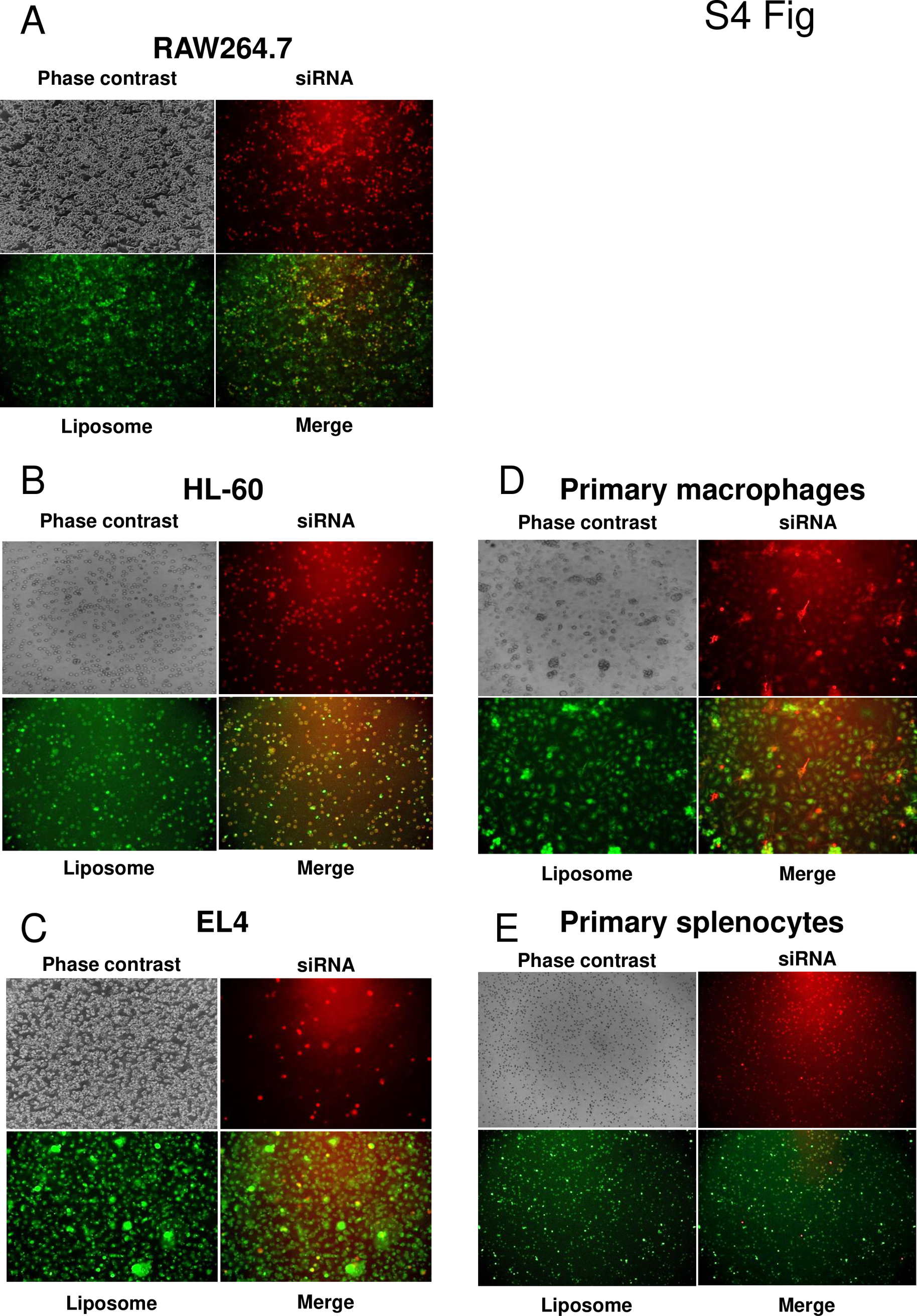

Supplement: S4 Fig — 2 × 10^6^ of different immune cell lines (A) RAW264.7, (B) HL-60, (C) EL4, (D) primary macrophages and (E) primary splenocytes were treated with LNP-S60 (10 μM) containing green fluorescent marker (Bodipy-FL) encapsulated siRNA (40 nM) coupled to red fluorescent probe (Cyanine-3). Cells were imaged 24 h post-treatment. (TIF) [file pone.0276905.s004.tif]

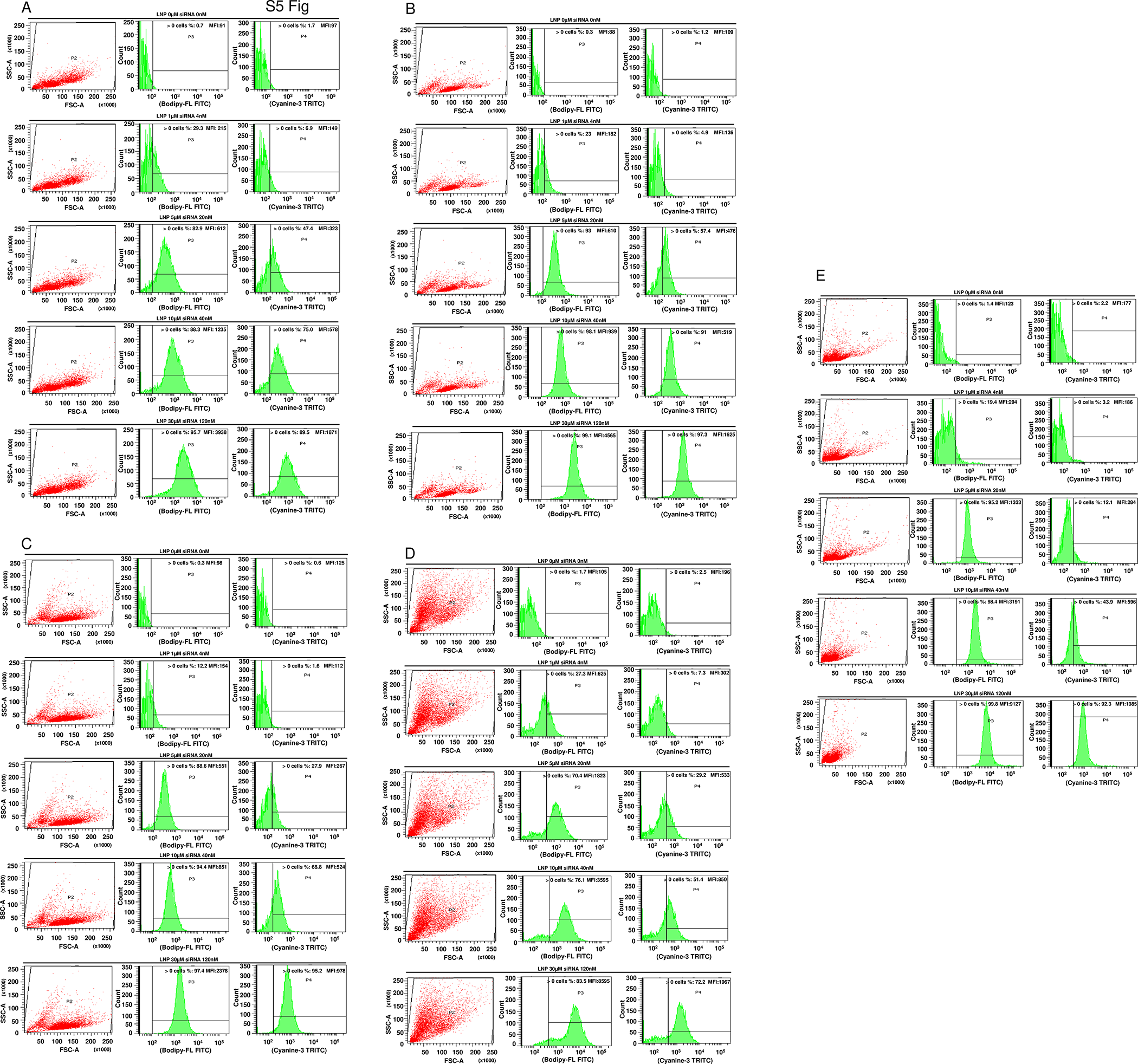

Supplement: S5 Fig — 2 × 10^6^ of RAW264.7 (A), HL-60 (B) and EL4 cells (C), or primary macrophages (D) or splenocytes (E), were seeded in 6-well plates in DMEM containing 10% FBS. On the next day, cells were treated/nontreated with 1 ml LNP-S60 (green fluorescent probe Bodipy-FL) at different concentrations containing fluorescent siRNA (red fluorescent probe cyanine-3) at 37°C in DMEM medium with 10% FBS. The cell population was firstly gated (P2 gate) based on Sideward and Forward scattering (SSC-A and FSC-A) and then the percentage of Bodipy-FL and cyanine-3 positive cells were measured in P3 and P4 gates, respectively. In addition, the mean fluorescence intensity (MFI) was also analyzed. Results are representative of three independent experiments. (TIF) [file pone.0276905.s005.tif]

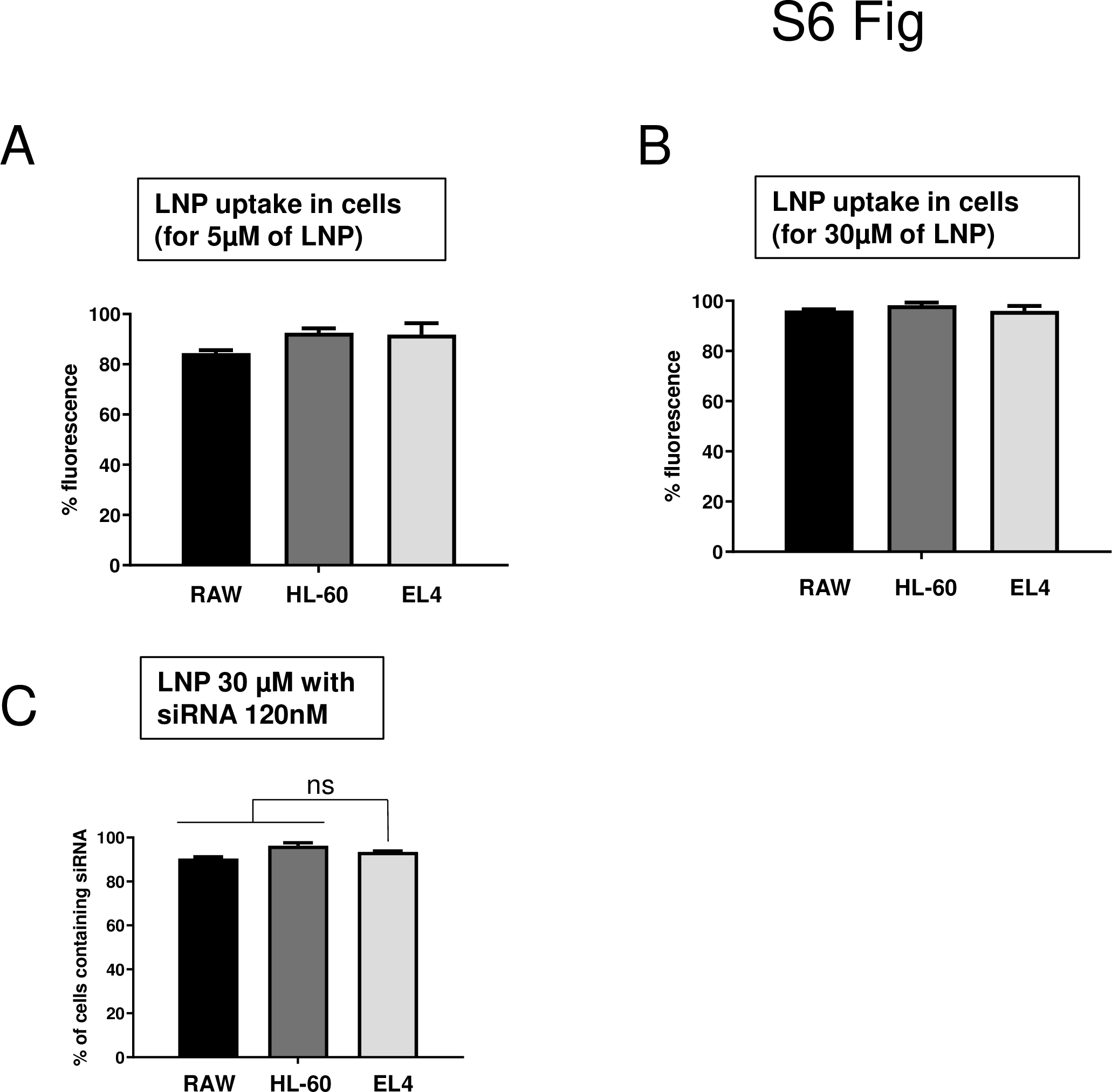

Supplement: S6 Fig — Different immune cell lines for macrophages (RAW264.7), neutrophils (HL-60), and lymphocytes (EL4) were transfected with LNP-S60 (green fluorescent probe Bodipy-FL) at a LNP concentration of either 5μM (A) or 30μM (B) for 24h. Then, the percentage of LNP-S60 positive cells was measured by flow cytometry. In addition, the percentage of siRNA positive cells after 24h of transfection with a LNP-S60 (30μM) armed with a siRNA (red fluorescent probe cyanine-3, 120nM) was also analyzed by flow cytometry (C). Results were represented by histograms. Results are representative of three independent experiments. mean ± SD were represented. ns, not significant; Two-tailed Mann-Whitney test was used for statistical analysis. (TIF) [file pone.0276905.s006.tif]

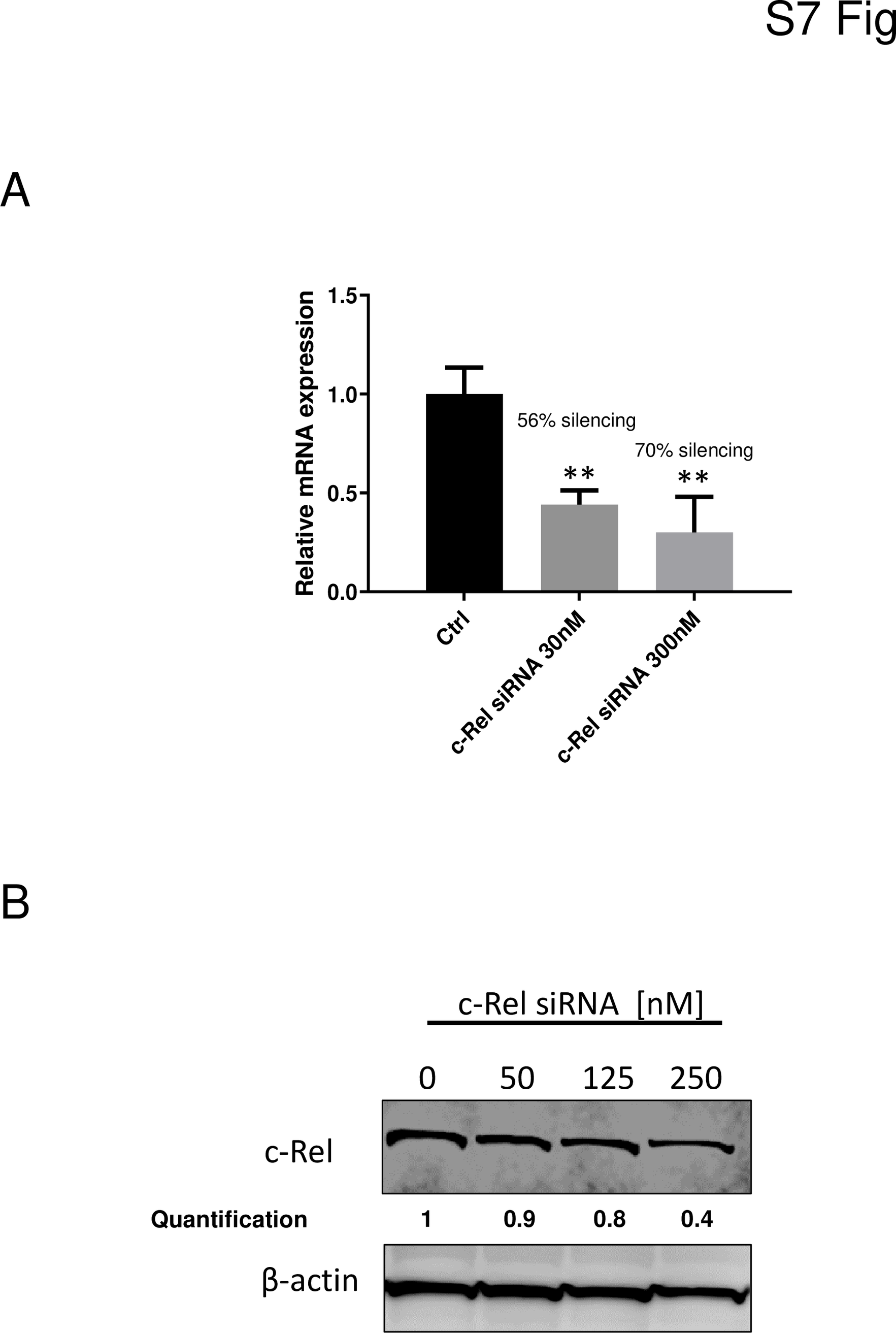

Supplement: S7 Fig — 2 × 10^6^ RAW264.7 cells were electroporated or not with different concentrations of murine c-Rel siRNA for 2 days. Changes in the levels of c-Rel mRNA (A) and protein (B) were determined. The levels of gene silencing were indicated in red, and results shown are representative of two independent experiments. Mean ± SD were represented. **, P <0.01; ns, nonsignificant. two-tailed unpaired t test was used for statistical analysis. The numbers between the blots (in blue) indicate the level of c-Rel expression relative to untreated cells. (TIF) [file pone.0276905.s007.tif]
