# Supplementary material for: Inhibition of c-Rel expression in myeloid and lymphoid cells with distearoyl -phosphatidylserine (DSPS) liposomal nanoparticles encapsulating therapeutic siRNA
Source: PLoS One. 2022 Dec 15;17(12):e0276905. doi: 10.1371/journal.pone.0276905 (PMC9754606; doi:10.1371/journal.pone.0276905)

Blot : Figure 5B

RAW264.7 cells

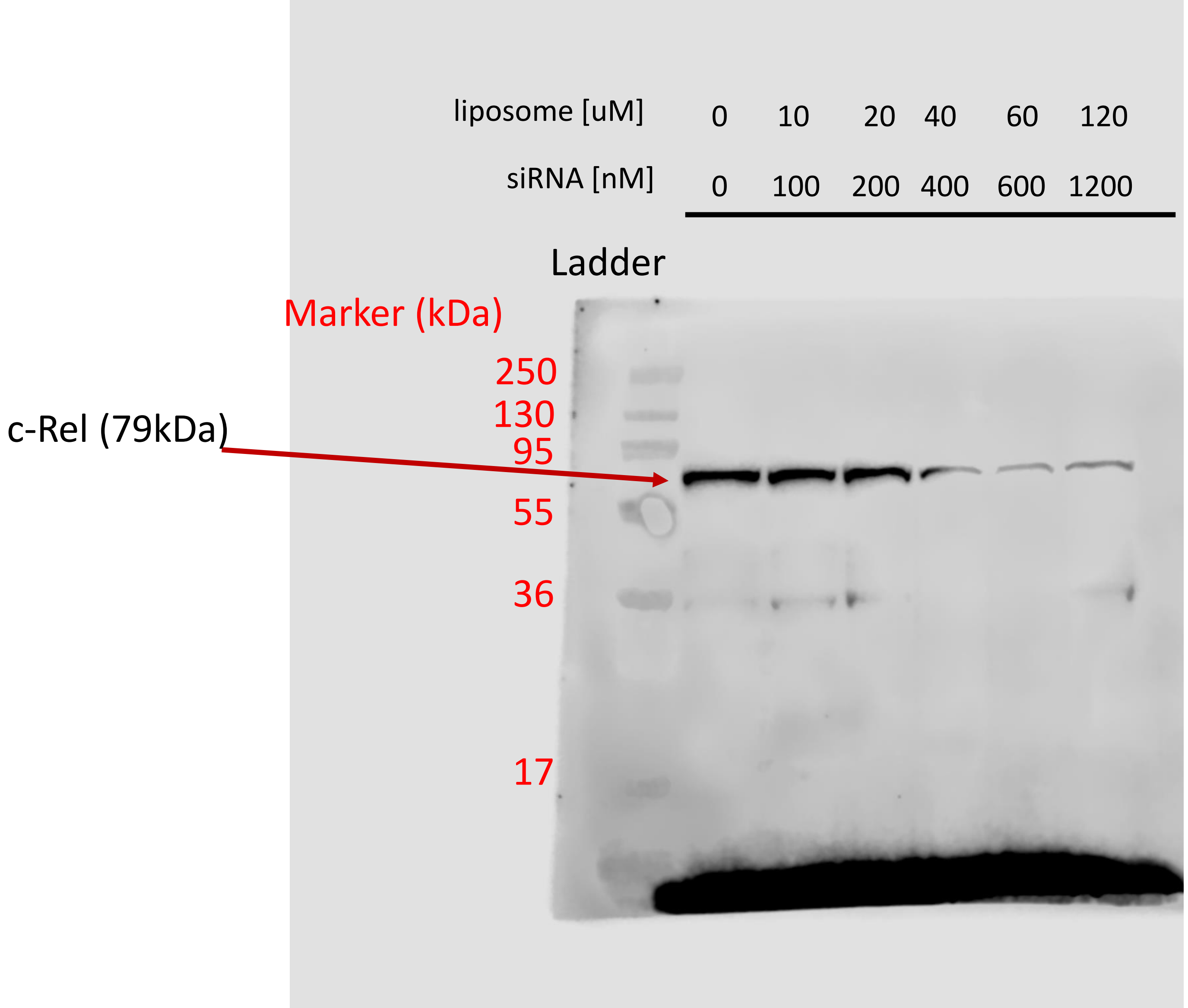

Blot : Figure 5B

RAW264.7 cells

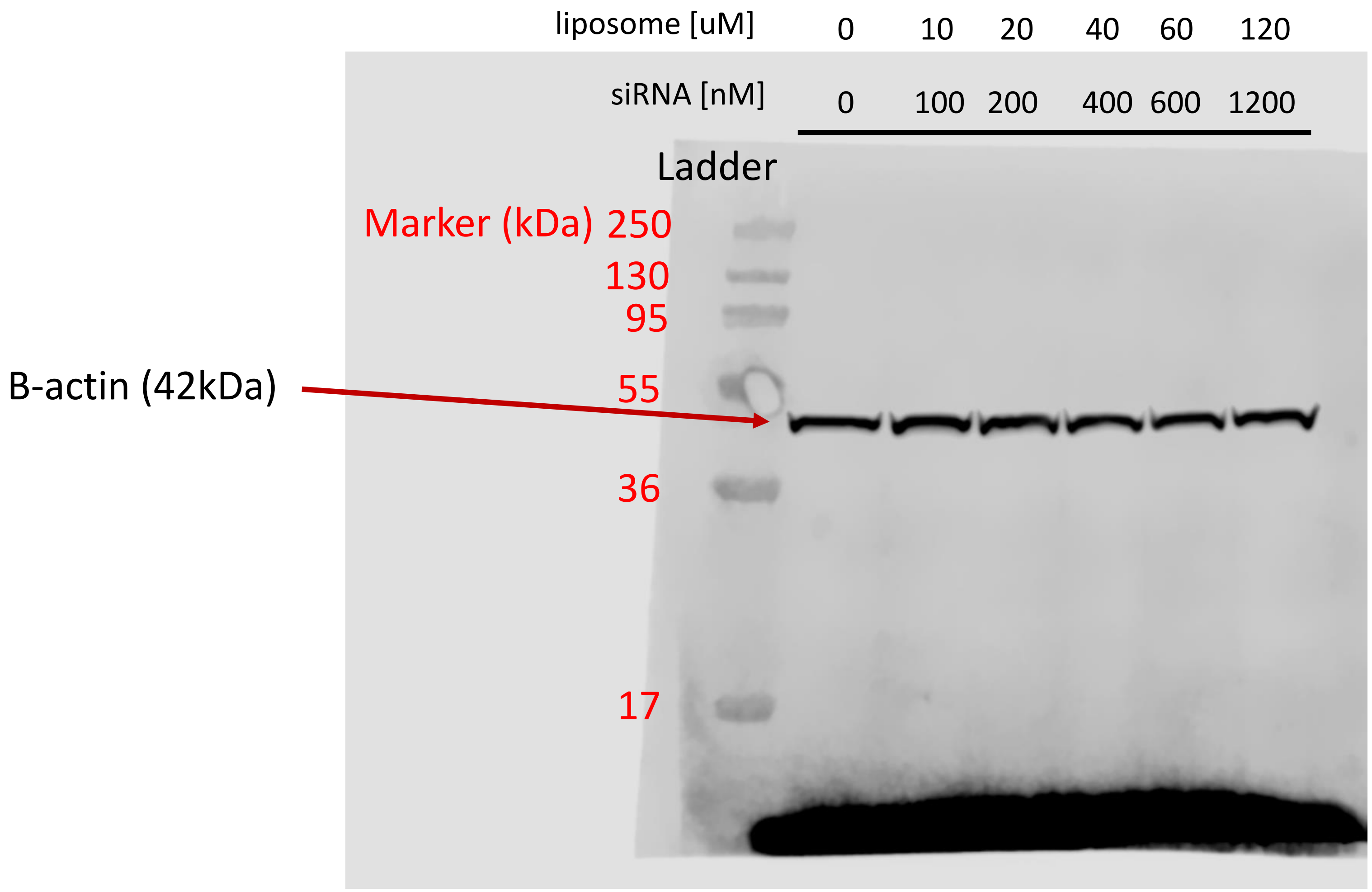

Blot : Figure 5D

HL-60 cells

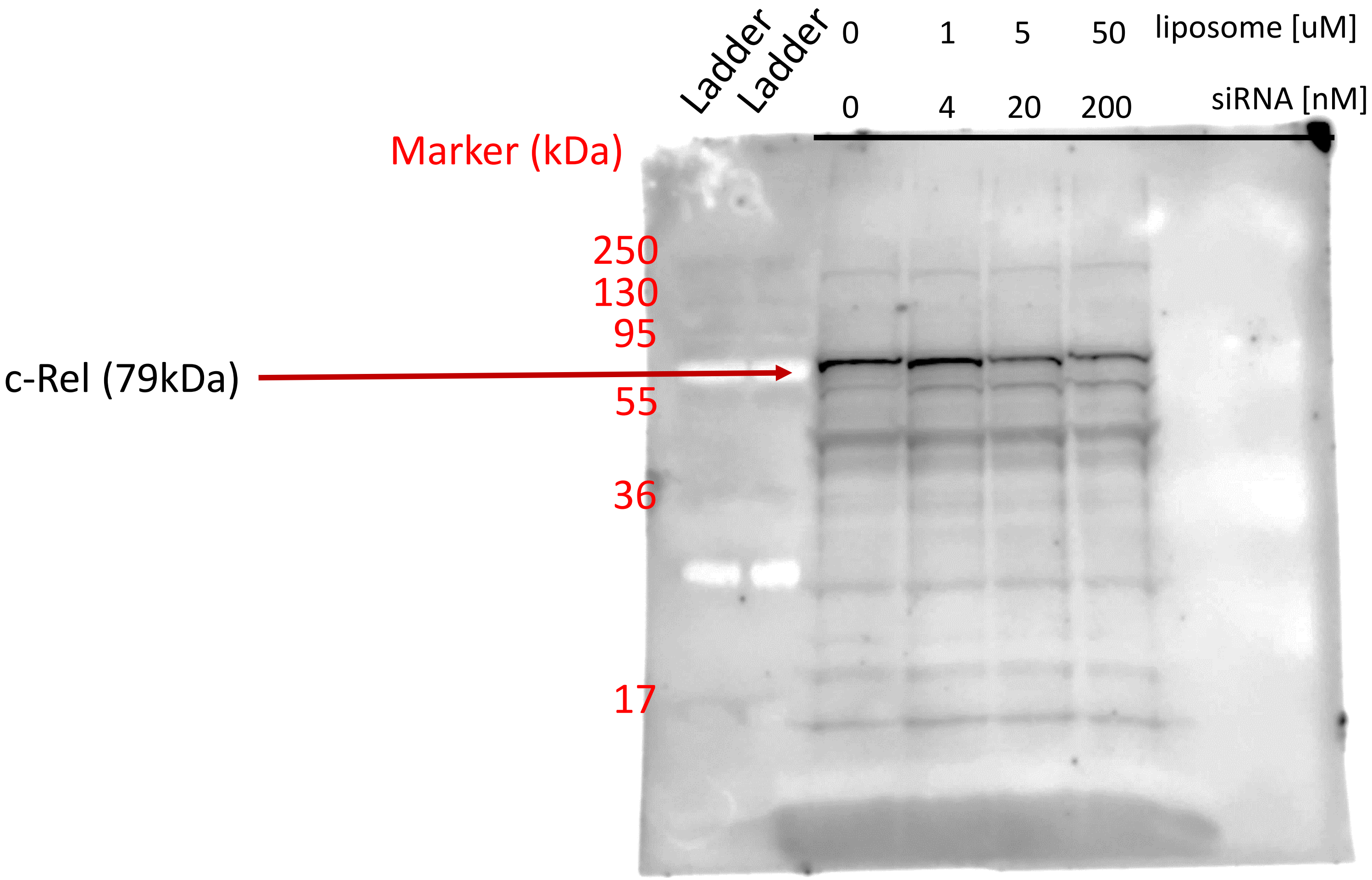

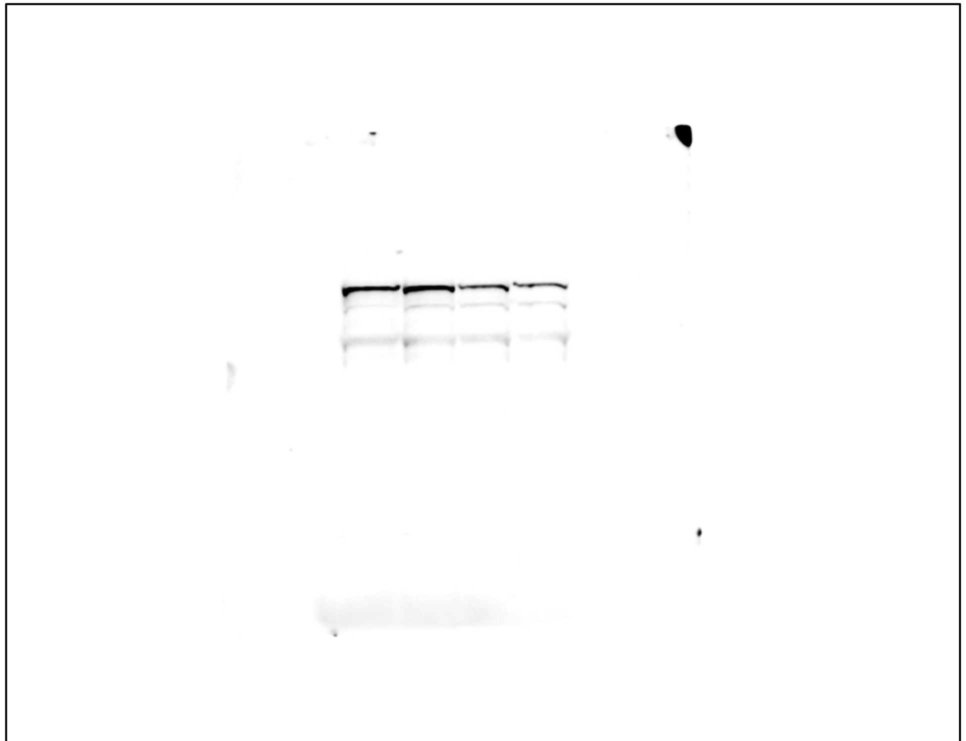

Same membrane with high luminosity

Blot : Figure 5D
HL-60 cells

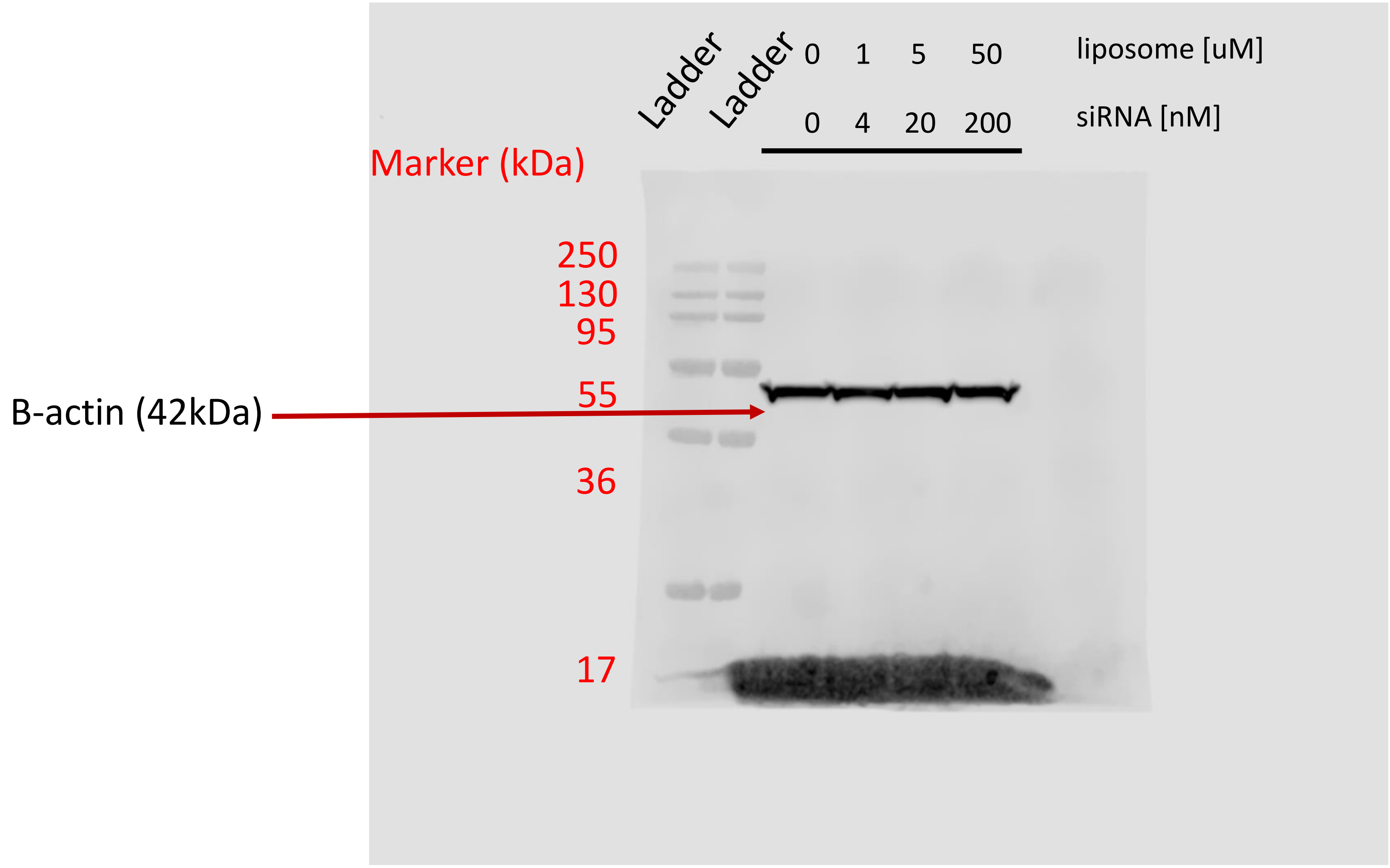

Blot : Figure 5 F

Primary macrophage

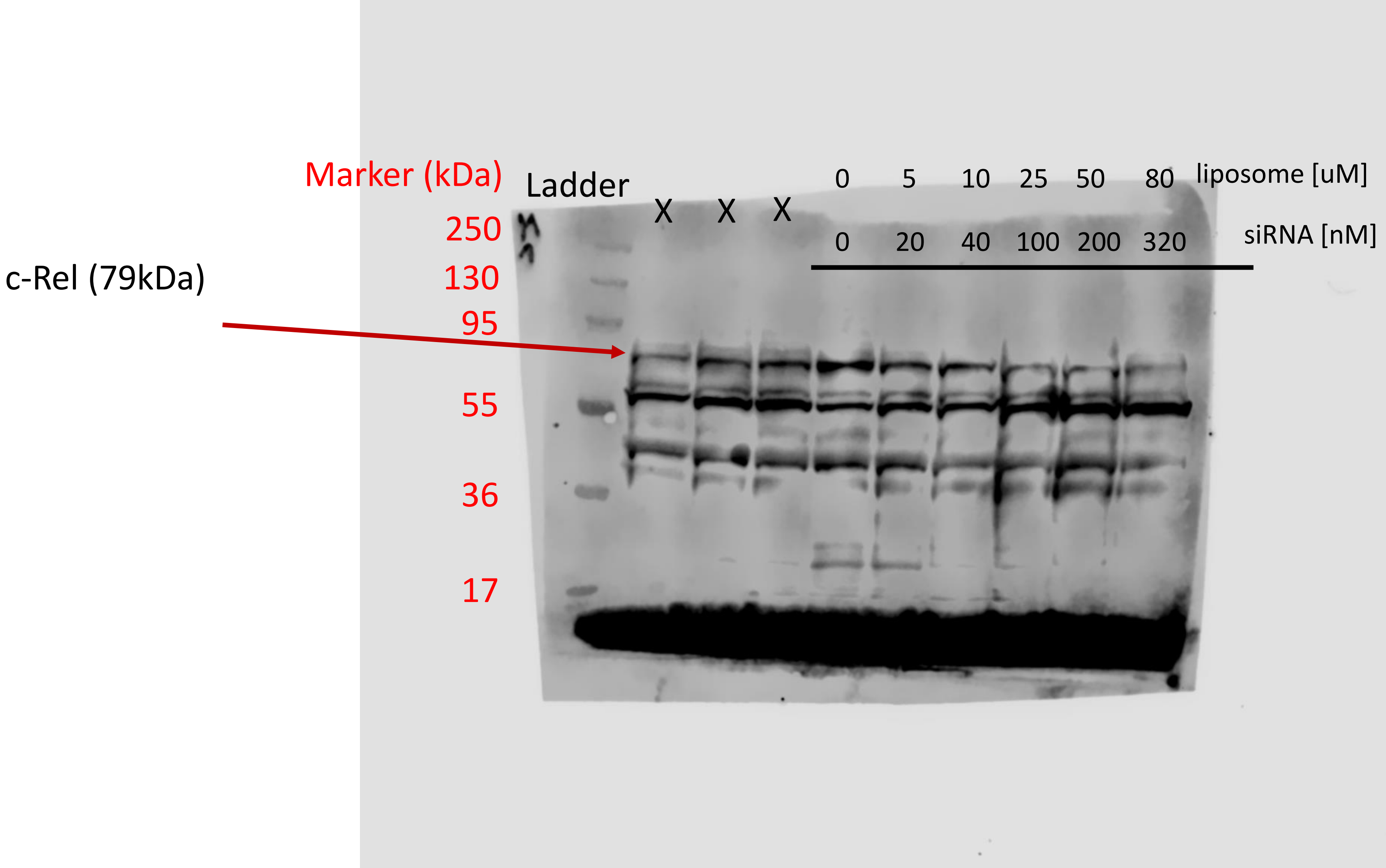

Blot : Figure 5 F

Primary macrophage

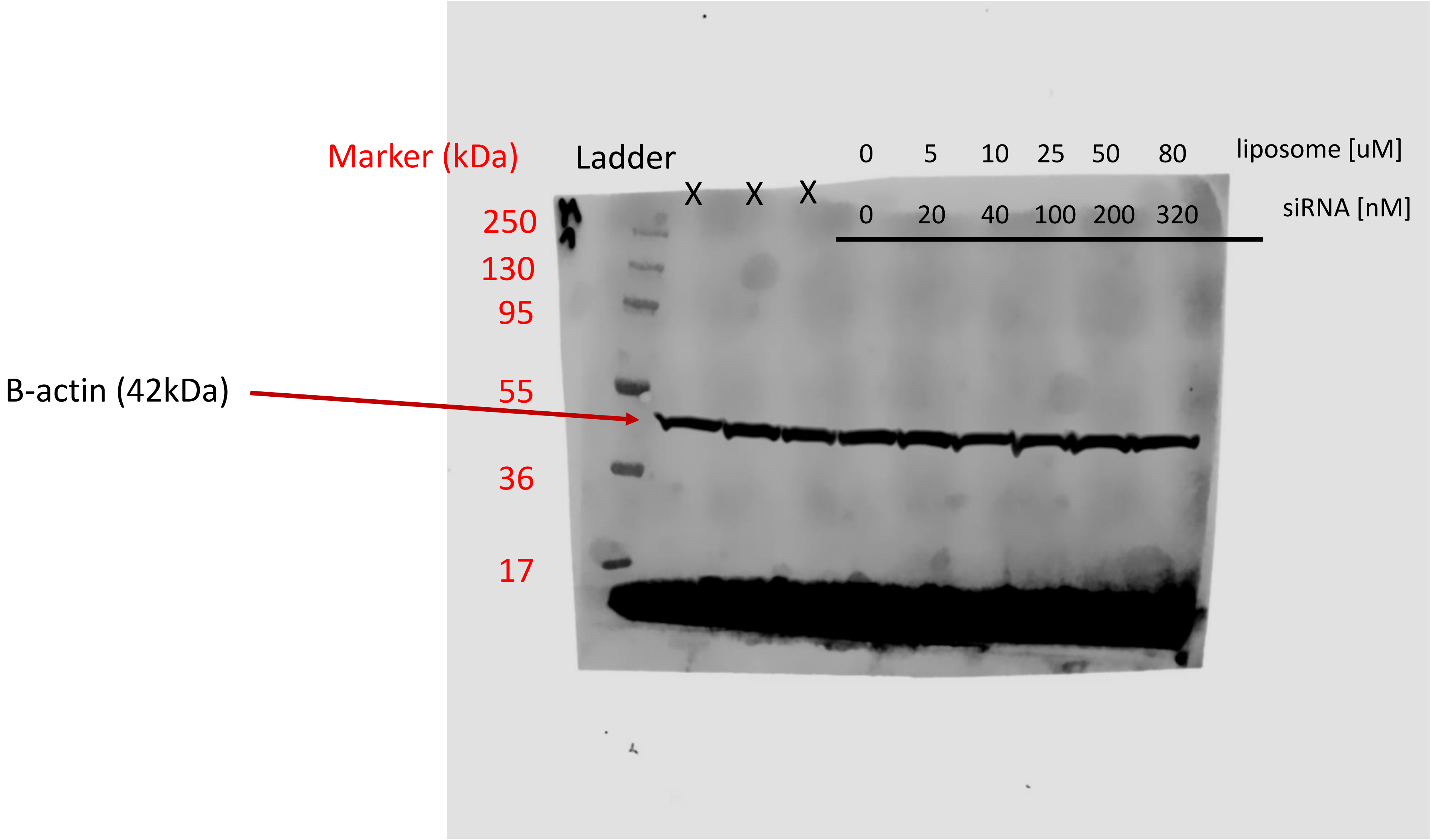

Blot : Figure 5 G

Primary macrophage

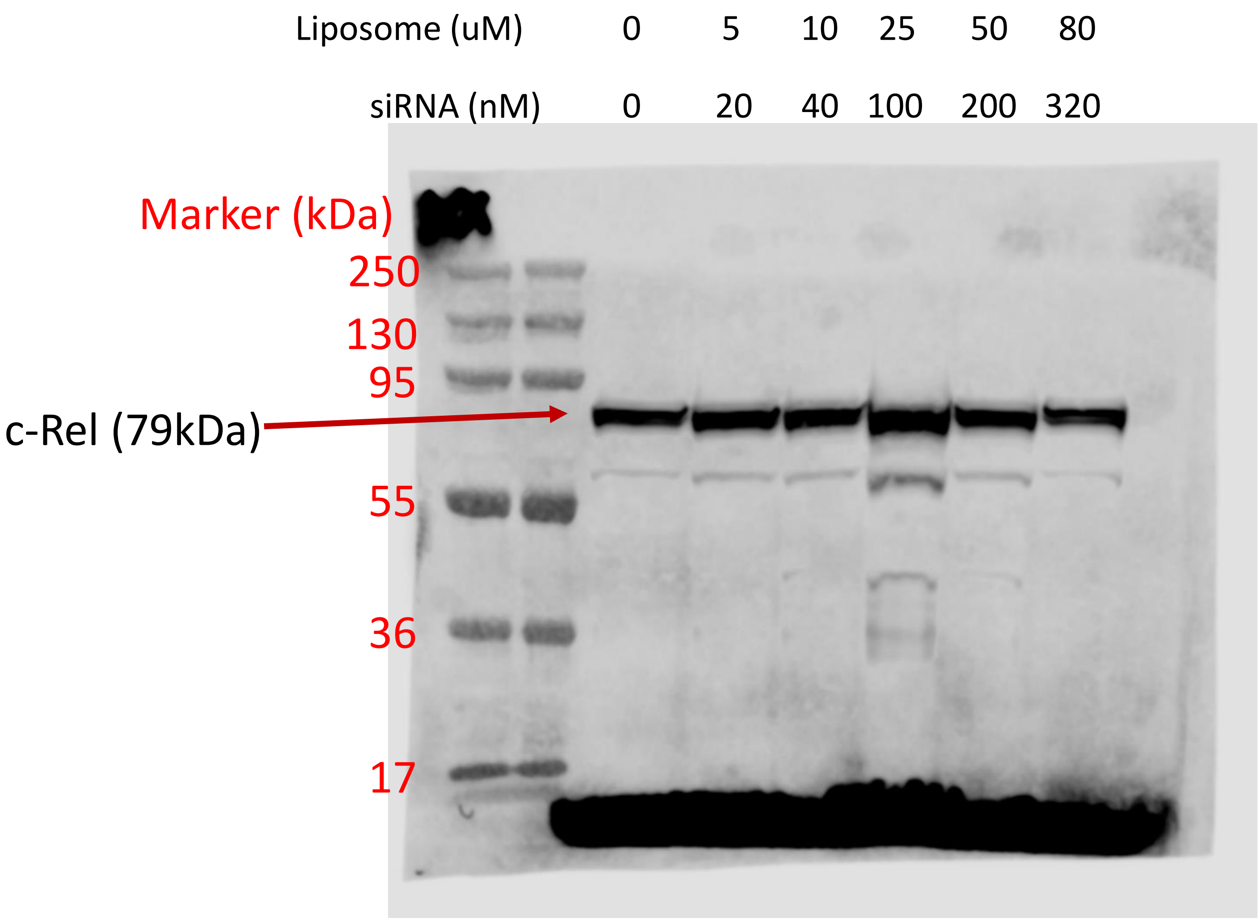

Blot : Figure 5 G
Primary macrophage

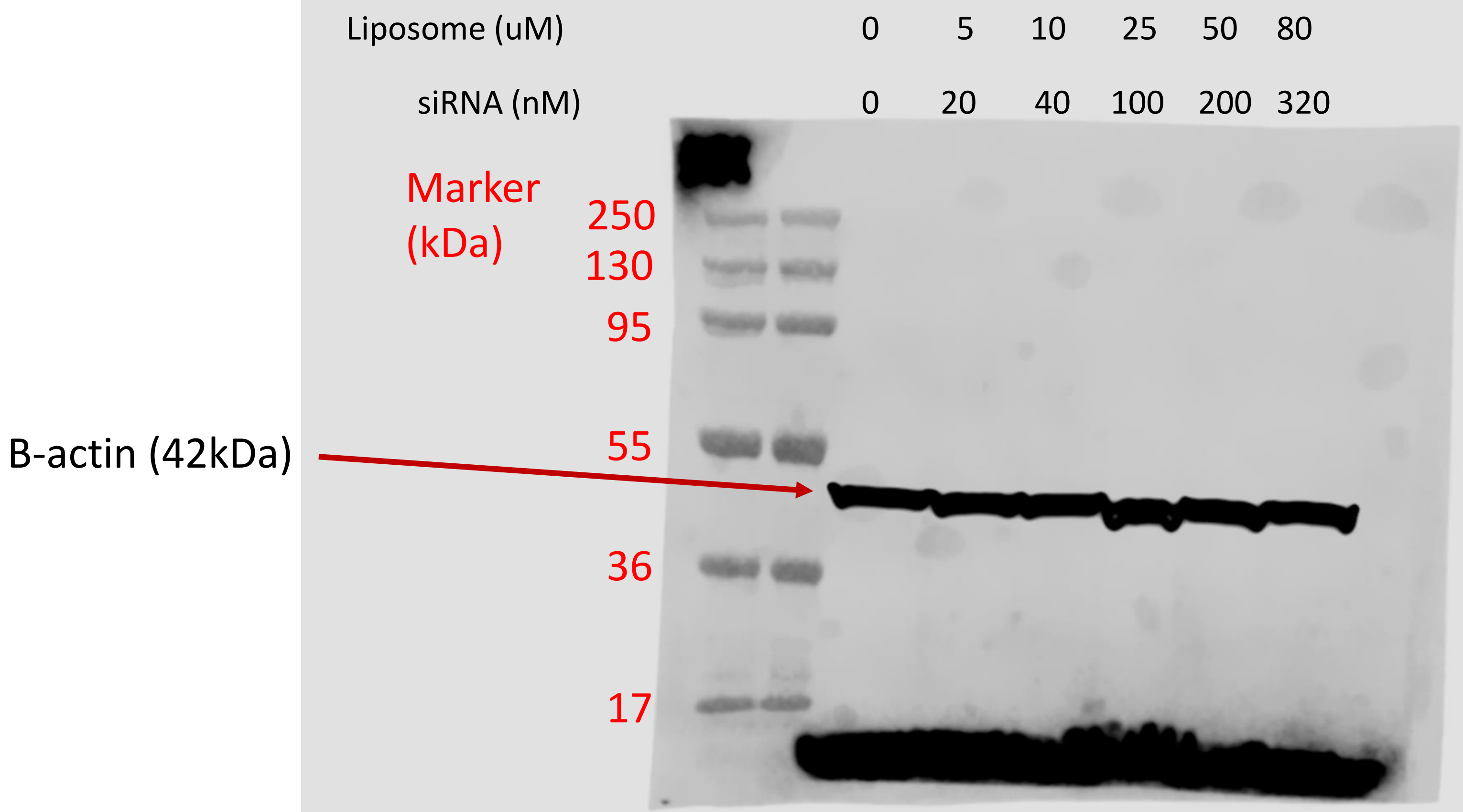

Blot : Supplementary Figure 7 B

RAW264.7 cells

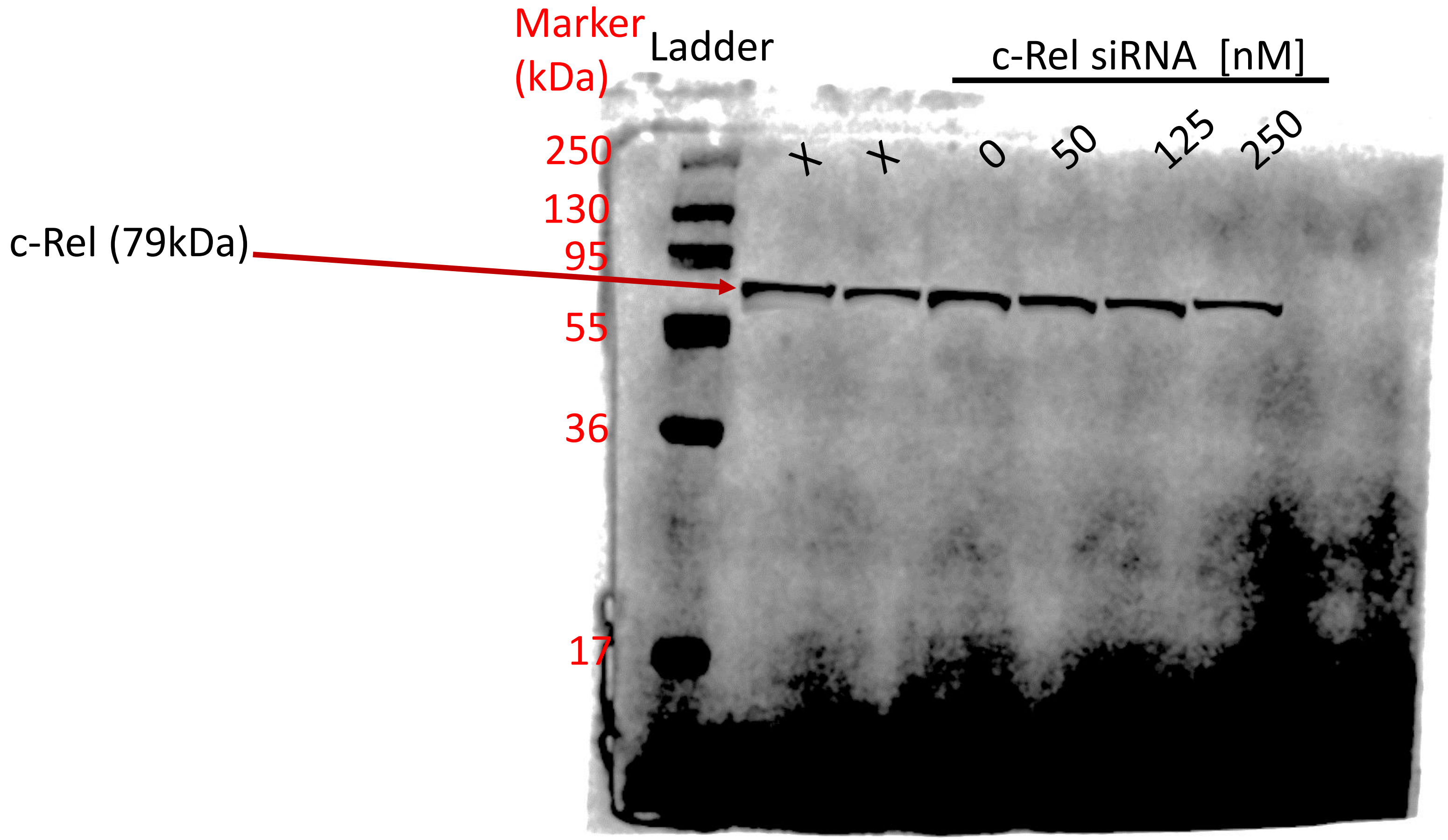

Blot : Supplementary Figure 7 B

RAW264.7 cells

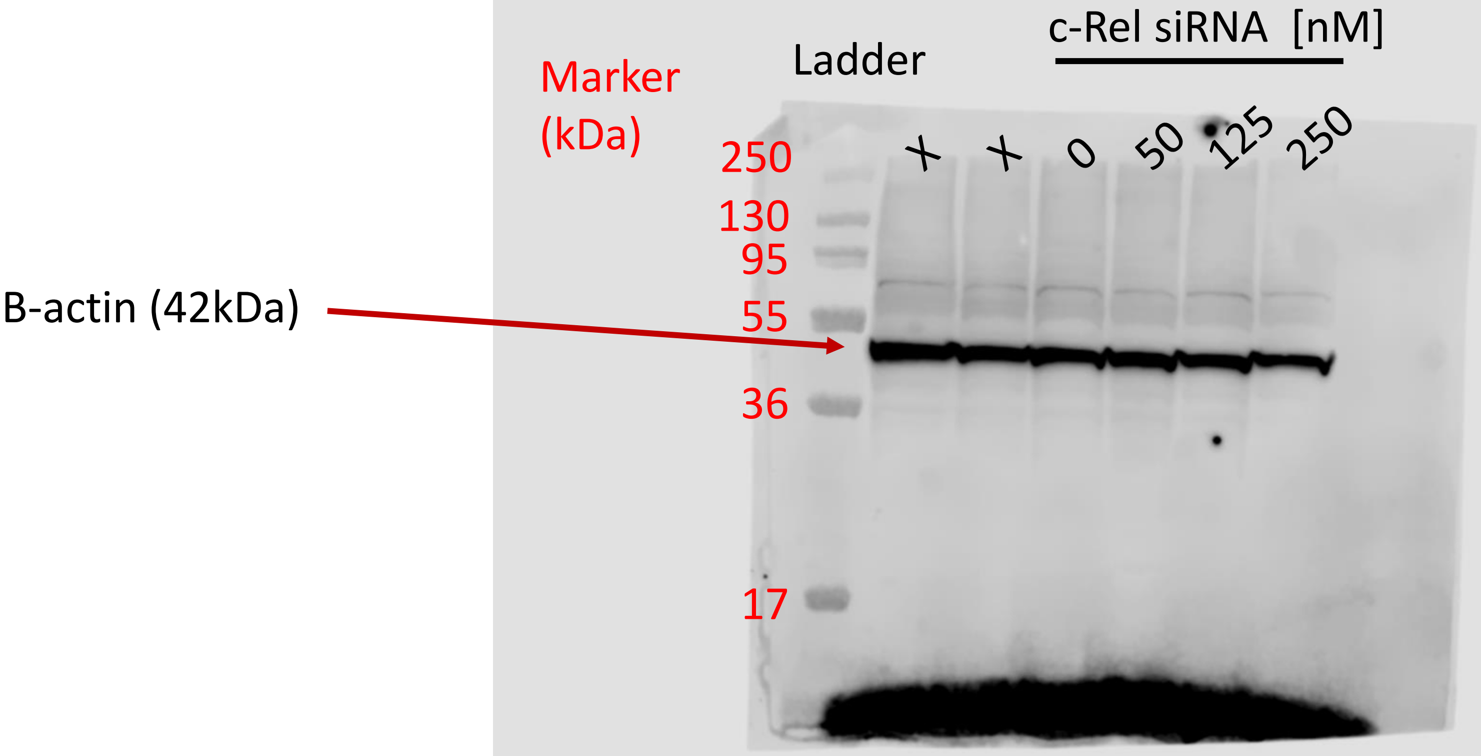

Supplement: S1 Raw images — (PDF) [file pone.0276905.s008.pdf]
